# LabWAS: Novel findings and study design recommendations from a meta-analysis of clinical labs in two independent biobanks

**DOI:** 10.1371/journal.pgen.1009077

**Published:** 2020-11-11

**Authors:** Jeffery A. Goldstein, Joshua S. Weinstock, Lisa A. Bastarache, Daniel B. Larach, Lars G. Fritsche, Ellen M. Schmidt, Chad M. Brummett, Sachin Kheterpal, Goncalo R. Abecasis, Joshua C. Denny, Matthew Zawistowski

**Affiliations:** 1 Department of Pathology, Northwestern University Feinberg School of Medicine, Chicago Illinois, United States of America; 2 Department of Biostatistics and Center for Statistical Genetics, University of Michigan, Ann Arbor, Michigan, United States of America; 3 Vanderbilt University Medical Center, Nashville, Tennessee, USA; Departments of Medicine and Biomedical Informatics, Vanderbilt University Medical Center, Nashville, Tennessee, United States of America; 4 Department of Anesthesiology, Michigan Medicine, University of Michigan, Ann Arbor, Michigan, United States of America; Institute for Molecular Medicine Finland (FIMM), FINLAND

## Abstract

Phenotypes extracted from Electronic Health Records (EHRs) are increasingly prevalent in genetic studies. EHRs contain hundreds of distinct clinical laboratory test results, providing a trove of health data beyond diagnoses. Such lab data is complex and lacks a ubiquitous coding scheme, making it more challenging than diagnosis data. Here we describe the first large-scale cross-health system genome-wide association study (GWAS) of EHR-based quantitative laboratory-derived phenotypes. We meta-analyzed 70 lab traits matched between the BioVU cohort from the Vanderbilt University Health System and the Michigan Genomics Initiative (MGI) cohort from Michigan Medicine. We show high replication of known association for these traits, validating EHR-based measurements as high-quality phenotypes for genetic analysis. Notably, our analysis provides the first replication for 699 previous GWAS associations across 46 different traits. We discovered 31 novel associations at genome-wide significance for 22 distinct traits, including the first reported associations for two lab-based traits. We replicated 22 of these novel associations in an independent tranche of BioVU samples. The summary statistics for all association tests are freely available to benefit other researchers. Finally, we performed mirrored analyses in BioVU and MGI to assess competing analytic practices for EHR lab traits. We find that using the mean of all available lab measurements provides a robust summary value, but alternate summarizations can improve power in certain circumstances. This study provides a proof-of-principle for cross health system GWAS and is a framework for future studies of quantitative EHR lab traits.

## Author summary

Electronic Health Records (EHRs) have emerged as an abundant data source for deriving phenotypes used in genetic association studies. EHRs provide a broad range of clinical data in large health system cohorts and are readily incorporated into large-scale meta-analyses. The abundance of available data in EHRs introduces unique technical challenges, particularly longitudinal clinical lab measurements which lack the structure of more commonly used disease diagnosis codes. Conflicting strategies exist in the literature and it is not clear how portable these strategies are across health systems. In this study we performed a proof-of-principle meta-analysis of 70 clinical lab traits in two large-scale health systems: BioVU from Vanderbilt University and the Michigan Genomics Initiative from Michigan Medicine. Despite the challenges of matching labs across the two health systems, we observed a high replication rate for known genetic variants. Further, we identified 31 novel associations, 22 of which replicated in an independent BioVU cohort, indicating the potential for future meta-analyses. Finally, we explored the impact of various analytic strategies, looking for consistent effects between our two cohorts, to determine optimal strategies for future genetic analysis of EHR-derived lab traits.

## Introduction

Laboratory testing is a key component of modern medicine. Laboratory measurements provide a glimpse into the functioning of the human body, allowing clinicians to diagnose and monitor disease. In most health systems, lab measurements are routinely captured in patient Electronic Health Records (EHRs) alongside disease diagnoses, free text notes and medical procedures to provide a detailed, longitudinal health history [1]. EHRs present exciting research potential by providing broad phenotyping on large cohorts with minimal cost [2,3].

Several large-scale genetic studies have already leveraged biobanks linked to EHRs, such as the UK Biobank [4], Japan Biobank [5], FinnGen [6] and HUNT [7], as sources of phenotypes for Genome-wide Association Studies (GWAS) [4–7]. The phenotypes are typically based on International Classification of Diseases (ICD) codes mapped to dichotomous traits [8]. Although disease is often thought of in all-or-nothing binary states, many diseases exist on a continuum with the ultimate clinical diagnosis occurring once a relevant quantitative laboratory measurement exceeds a pre-determined threshold. For example, hypercholesteremia, diabetes mellitus and chronic kidney disease are each diagnosed almost entirely on measurements of low density lipoprotein (LDL), glycated hemoglobin (or glucose) and creatinine, respectively. Laboratory measurements can therefore be a more sensitive measure of underlying health than diagnosis and may provide a more powerful outcome for analysis. As an example, the hypercholesterolemia and coronary artery disease risk locus *PSCK9* was initially discovered based on quantitative LDL measurement rather than clinical diagnosis [9,10]. In contrast to binary disease phenotypes, there are fewer examples of genetic analyses of EHR-derived quantitative lab values [11–13]. Hereafter, we use the term lab traits to refer to quantitative biomarkers assayed through clinical laboratory testing (e.g., “creatinine", "LDL cholesterol"), and the term lab measurements to refer to realized values of these tests stored in patient EHRs.

The rich source of quantitative lab measurements in EHR cohorts comes with unique concerns. Quantitative traits collected specifically for research purposes typically use a controlled experimental design to ensure consistency among samples. In contrast, lab measurements contained in EHRs are a historical record of medical care. As such, patients may have hundreds of lab measurements for some traits and none for others, depending on their specific health history and utilization of the health system. The measurements can be collected in times of sickness or good health leading to substantial variation in measurements for the same lab. Lab measurements can also be artificially modified by prescription medication, such as statin use for lowering LDL cholesterol. Moreover, recruitment mechanisms and health system demographics can dramatically shape the overall health of the biobank cohort, which in turn dictates lab measurements available for analysis. The broad impact of using such “real world” measurements for genetic association studies is unclear. Questions remain over the effect and robustness of analytic choices made when analyzing EHR-based lab traits including how best to summarize complicated, longitudinal lab measurements and whether comorbid diseases highly correlated with lab measurements must be considered. Prior studies are not consistent in addressing these concerns. For example, GWAS of EHR-derived quantitative traits in Biobank Japan enrolled patients with at least 1 of 47 diagnoses and controlled for all 47 diagnoses while testing each lab [11]. In contrast, an analysis of labs within the Geisinger EHR did not control for underlying disease states [14]. The variety of methods to summarize lab measurements and models to test for genetic association indicates that the question of how to analyze these data remains unsettled.

In this paper we explore strategies for analyzing quantitative lab measurements extracted from EHRs and describe the first large-scale meta-analysis of EHR-derived lab traits across independent health systems. We used lab measurements and genetic data from two academic health systems: the BioVU cohort from Vanderbilt University [15] and the Michigan Genomics Initiative (MGI) from Michigan Medicine [16]. Meta-analysis offers a mechanism to increase sample size and power for detecting genetic risk variants but comes with distinct challenges for EHR lab traits, particularly matching lab traits between health systems and determining specific analysis protocols. The cohorts differ dramatically in their recruitment mechanisms, patient composition and recording format for lab measurements: MGI was predominantly recruited through inpatient surgical encounters at Michigan Medicine whereas BioVU recruitment required outpatient appointments at Vanderbilt University Medical Center. As a result, MGI is enriched for diseases treated surgically such solid tumors [16]. This heterogeneity reflects the reality of EHR-based phenotyping, and strategies must be developed for future collaborative work on the growing number of EHR-linked biobanks.

Our initial challenge was identifying which labs to meta-analyze between the health systems. Accurately matching labs is complicated by the fact that no standardized coding scheme exists for lab measurements. Dichotomous disease traits are readily matched between health systems using the ubiquitous ICD coding system for disease diagnoses [17]. Although the Logical Observation Identifiers Names and Codes (LOINC) system offers the promise of interoperability for lab traits, it is cumbersome and maps poorly onto other ontologies [18]. For example, there are 21 distinct codes for blood glucose which might not be used consistently between institutions. Moreover, health systems may adopt their own idiosyncratic internal terminology for electronic recording of lab results. Based on a methodical manual review of EHR text descriptions and lab measurements, we identified 70 lab traits between BioVU and MGI that could be matched with high confidence. We extracted previously identified variants for these lab traits from the GWAS catalog to serve as true positive variants for assessing subsequent analyses. Our meta-analysis replicated nearly 75% of these true positive variants, validating both the accuracy of lab matches across health systems and the overall quality of the EHR lab data. Further, we discovered 31 novel lab-associated variants across 22 labs, including the first reported associations for the saliva and pancreatic enzyme amylase and bicarbonate CO2, a gaseous waste product from metabolism carried in the blood. We immediately replicated 22 (71%) of these novel associations using an independent second set of BioVU samples.

The meta-analysis required several strategic choices regarding data preparation and statistical analysis. We explored the consequences of various analytic choices using a series of mirrored analyses performed in MGI and BioVU. In particular, we varied the summary statistic for lab measurements and the inclusion of covariates to control for comorbid diseases in the GWAS. We compared the results between the independent biobank cohorts to assess consistency of effects. We hypothesized that alternative summary statistics to the basic mean could provide more powerful genetic analyses. We considered: the median lab measurement due to robustness against data recording errors and extreme measurements, the first available lab measurement to mitigate the effects of prescription drugs on modifiable lab traits, and the maximum recorded measurement to magnify variation in extreme measurements. The comorbidity analysis compared GWAS results from models that included indicator covariates for a wide array of diseases to models that did not.

The complete set of GWAS summary statistics from this analysis are broadly available to the research community. We encourage others to use this data to replicate their own GWAS findings and perform hypothesis-driven lookups on specific SNPs or lab traits of interest. Our results are viewable through an interactive *PheWeb* web browser [19] at http://pheweb.sph.umich.edu/mgi-biovu-labs and available for bulk download at https://phewascatalog.org/labwas and ftp://share.sph.umich.edu/mgi_biovu_labwas/.

## Methods

### Datasets

We analyzed data from two university hospital biobanks that link electronic health records with genetic data: BioVU from Vanderbilt University and the Michigan Genomics Initiative (MGI) from Michigan Medicine. We restricted our analysis to unrelated patients of European ancestry because of insufficient patient sample sizes and a paucity of known variants in non-European populations.

The BioVU cohort has been described previously [15]. Briefly, DNA was extracted from surplus blood samples and genotyping data was linked to de-identified EHR data. For this study, we used a cohort of 20,515 individuals genotyped on the Multi-Ethnic Genotyping Array (MEGA) from Illumina and estimated to be of European ancestry by admixture [20]. We included 843,242 SNPs that passed standard marker QC filters and had a minor allele frequency >1%. We retrieved all available lab measurements in this cohort that occurred when the subject was at least 18 years of age.

The MGI cohort has also been described previously [16]. Briefly, MGI samples were recruited primarily through surgical encounters at Michigan Medicine and provided consent for linking of their EHRs and genetic data for research purposes. MGI samples were genotyped on customized Illumina HumanCoreExome v12.1 bead arrays. European samples were identified using Principal Component Analysis. We used a data freeze consisting of 37,354 unrelated European individuals for this analysis. MGI samples were imputed to the Haplotype Reference Consortium using the Michigan Imputation Server [21], providing ~14 million SNPs with a minimac imputation quality R2>0.3 and an allele frequency greater than 1e-6. We analyzed the set of ~800K overlapping SNPs between the MGI imputed genotypes and the BioVU MEGA array for this study.

### Harmonization of labs between health systems and the GWAS Catalog

We extracted all available clinical lab measurements and metadata from the electronic health records of MGI samples and BioVU samples. We collapsed distinct labs when obvious duplications were present (e.g., “Eosinophils” and “EOSINOPHILS”). Available metadata differed slightly between the health systems but included brief text descriptions, unit of measurements, and range for normal values. We excluded individual lab measurements taken outside the health system labelled as “External.” In cases where multiple tests examined the same analyte, e.g. blood glucose, we removed point of care (POC) tests which are more susceptible to technical artifacts and tend to be deployed in intensive care or emergency settings where acute disease or treatment effects supervene determinants of the underlying baseline [22,23]. Lab traits were matched between the Vanderbilt and Michigan health systems based on manual curation of the metadata including recorded lab names, clinical descriptions, measurement units, range of measurements, and patient count.

### Disease phenotypes

In order to study the effect of underlying health conditions we extracted ICD9 and ICD10 diagnosis codes from the EHR of the BioVU and MGI cohorts. We searched for diagnosis for 42 diseases with the potential to alter a clinical lab measurement (S1 Table). We started with the disease list used in the BioBank Japan lab analysis [11] and removed diseases which do not occur in our population (e.g. febrile seizures of infancy) and those expected to have minimal effect on labs (e.g. cataracts). We supplemented their list with chronic diseases expected to have a large impact on labs due to their prevalence (e.g. hypertension). We created an indicator variable for each disease (1 if the sample had at least one qualifying ICD code for the specific disease and a 0 otherwise) to include as covariates in GWAS regression analyses.

### Statistical analysis

#### Intra-cohort Genome-wide Association Studies

We first performed GWAS analysis of each lab trait separately in the MGI and BioVU cohorts. We performed multiple GWAS for each lab, varying the statistic used to summarize the longitudinal lab measurements for each sample (mean, median, first available measurement and maximum available measurement) and the inclusion of binary indicators for diagnosis comorbid diseases in the GWAS regression.

For each GWAS, the distribution of lab summary statistics was inverse normalized separately within the MGI and BioVU cohorts prior to regression analysis. In a separate analysis of the BioVU cohort, we determined that inverse normalization of lab values performed better than applying no transformation, or a log or square root transformation for controlling GWAS type I error. Genome-wide association tests were performed on the inverse normalized traits using additive linear regression models containing age, sex and four principal components as covariates. The comorbidity model controlled for disease status by inclusion of an additional 42 covariates for the binary disease phenotypes. The regression analyses were performed in the BioVU cohort using PLINK [24] and in the MGI cohort using *epacts 3*.*3*.*0* [25].

#### Comparison of p-values across cohorts

**Fig 1:**
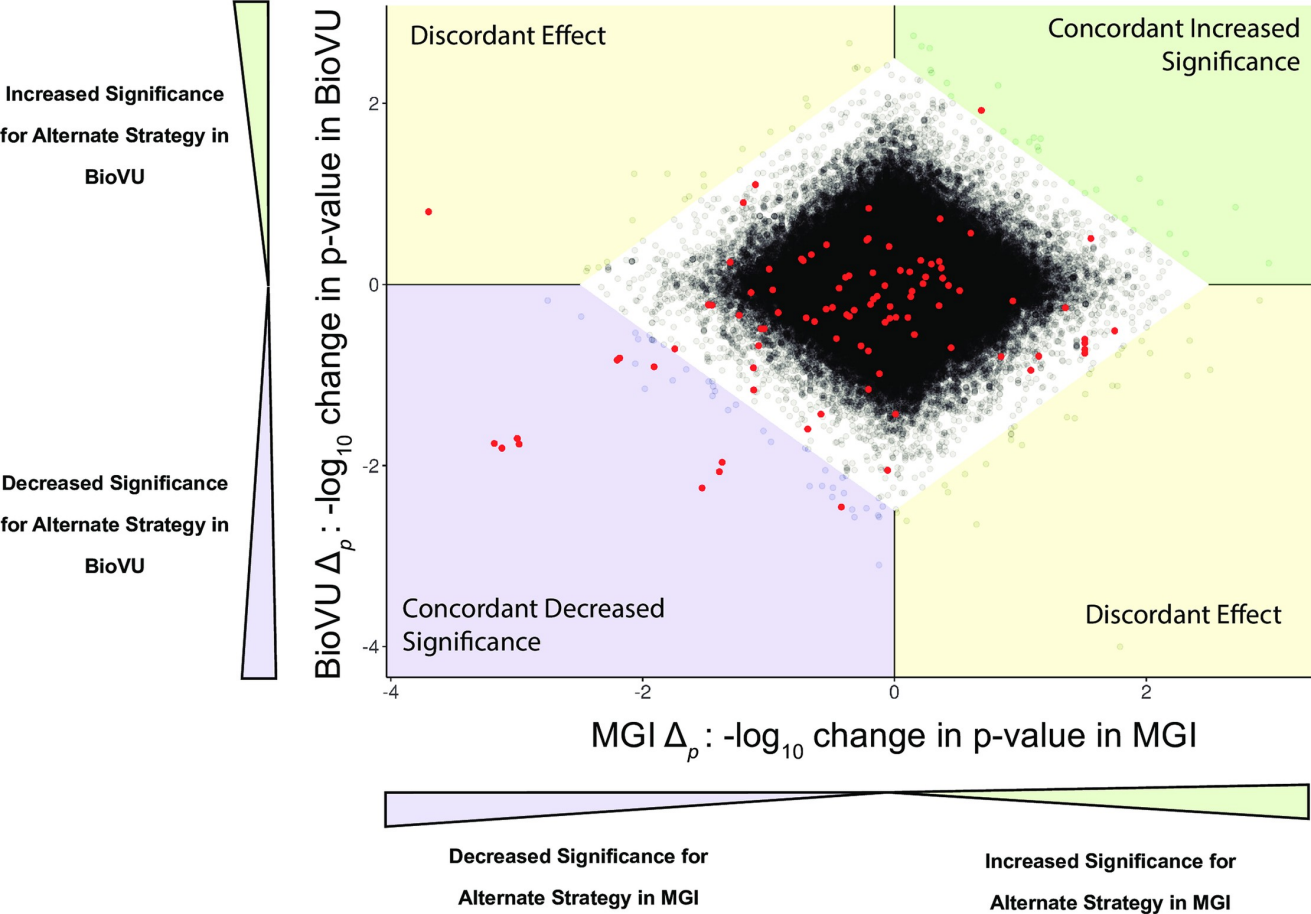
Scatterplot of Δ_*p*_ in MGI and BioVU when using the first available measure rather than the mean measurement in a GWAS of Cholesterol level. Δ_*p*_ is the -log fold change in p-value at a SNP for using an alternate analysis, in this case the first available lab measurement. Each dot is a SNP, with red dots indicating GWAS catalog SNPs for the specific lab trait. The white diamond contains 99.9% of SNPs and is used to identify SNPs with the largest changes in p-value due to the alternate analysis. SNPs outside the bounding diamond in the top right (green) quadrant show a concordant increase in significance in both MGI and BioVU, that is, SNPs for which the alternative strategy increases significance in both cohorts. Conversely, SNPs in the bottom left (blue) quadrant show a concordant decrease in significance in both MGI and BioVU. SNPs in either the top left or bottom right (yellow) quadrants have a discordant effect, indicating a large increase in p-value in one cohort but a large decrease in p-value in the second cohort. In this example, one catalog SNP showed a concordant increase in significance when using the first available lab measure, 11 catalog SNPs had a concordant decrease in significance and one SNP had discordant effects. The complete set of scatterplots for each analyzed lab and alternative analysis strategy (summary statistic and comorbidity model) are included in the S1 Fig. Tables 3 and 4 summarize the movement of catalog SNPs for each lab and analysis strategy.

We treated the GWAS of mean trait value with no disease covariates as the default. We quantified the impact of each alternate analysis strategy relative to the default analysis by computing the log fold change in p-value between the alternative and default analysis for each analyzed SNP. That is, for each SNP we compute the quantity

$$\Delta_{p}=-\log_{10}\left(p‐value\;for\;alternative\;analysis/p‐value\;for\;default\;analysis\right)$$

for the MGI analysis and the BioVU analysis separately. A positive value of Δ_*p*_ indicates a SNP that increases in significance (smaller p-value) for the alternate summary statistic. A negative value of Δ_*p*_ indicates a decrease in significance for the alternate analysis. Scatterplots of Δ_*p*_ computed in MGI and BioVU summarize the magnitude and consistency of change in p-value significance between the cohorts (Fig 1, S1 Fig). We performed LD-pruning on non-catalog SNPs to simplify the scatterplots. Since most SNPs are not associated with the lab trait of interest, alternative summarizations simply result in independent noise between the two cohorts, resulting in a diamond shaped pattern centered at the origin.

We implemented a heuristic to formally distinguish the SNPs with largest changes in p-value between the alternative and default analysis methods from those with movement due simply to random noise. The heuristic generates a bounding quadrilateral polygon around the diamond cluster of points, generated using simulated annealing to determine the bounding coordinates of a polygon containing 99.9% of all SNPs. We defined SNPs outside the boundaries of the polygon as those with largest simultaneous changes in p-values in both cohorts. Catalog SNPs located outside the bounding polygon were classified as having either a concordant increased effect if p-value significance increased in both MGI and BioVU, a concordant decrease effect if p-value significance decreased in both MGI and BioVU or a discordant effect if the p-value increased in significance in one cohort but decreased in the other.

#### Meta-analysis

We meta-analyzed the GWAS results from the MGI and BioVU default analysis (mean trait value, no disease covariates). The meta-analysis was performed using *METAL* by combining study-specific GWAS effect size estimates and standard errors [26]. We computed genomic control inflation factors (*λ*_GC_) on a set of LD-pruned SNPs for each meta-analyzed lab.

#### GWAS catalog variants

We created a list of previously identified genetic associations for each analyzed lab trait using the GWAS catalog [27] (downloaded 9/27/2017). We searched the catalog for quantitative phenotypes matching our analyzed labs using pattern matching in the DISEASE_TRAIT, MAPPED_TRAIT, and P_VALUE_TEXT columns. We searched for each lab using multiple potential string patterns, for example “AST”, “aspartate aminotransferase”, “SGOT”, and “serum glutamine oxaloacetic aminotransferase”. For purposes of replication, we limited our catalog search to studies of European cohorts performed on adults of both sexes without disease-based sampling (e.g. glucose measurements in type 2 diabetes samples) and required a reported p-value of at least 5e-8. We considered a catalog association replicated if the meta-analysis p-value for our corresponding lab was < 0.05 and the BioVU and MGI studies had the same direction of effect.

#### Definition of novelty

We report novel lab-SNP associations as those reaching genome-wide significance that have not been previously reported in European populations and are not reasonably expected based on existing SNP-lab associations in similar labs. We used the following criteria: meta-analysis p-value <5e-8, consistent direction of effect between MGI and BioVU and at least 1 megabase from any previously reported SNP for the given lab or a related lab in the GWAS catalog. Here, we define related labs as those which are commonly ordered as part of a panel of correlated tests (e.g. AST and ALT for liver function) or arithmetically-dependent traits (e.g. LDL and total cholesterol), and therefore likely to indicate the same biological association. We report the “peak” or most significant SNP when a group of novel SNPs are in linkage disequilibrium.

#### Replication of novel associations

We performed a replication analysis of novel associations identified in the meta-analysis using an independent cohort of BioVU samples that became available after the original meta-analysis was performed. This replication cohort consisted of 29,043 European ancestry adult individuals with extant lab data recruited using the same procedure as the initial BioVU cohort, genotyped on the same MEGA genotyping array, and subjected to the same data QC procedure. We declared a novel association to be replicated if the replication p-value was <0.05 and the direction of effect was consistent with that from the meta-analysis.

### Ethics statement

Data were collected according to Declaration of Helsinki principles. MGI study participants’ consent forms and protocols were reviewed and approved by the University of Michigan Medical School Institutional Review Board (IRB ID HUM00099605 and HUM00155849). Opt-in written informed consent was obtained for each MGI participant. BioVU is Vanderbilt University's biobank of DNA extracted from leftover and otherwise discarded clinical blood specimens. BioVU operates as a consented biorepository; all individuals must sign the BioVU consent form in order to donate future specimens.

## Results

We extracted all available clinical lab measurements from the electronic health records (EHRs) for genotyped samples in two academic biobank cohorts: the Michigan Genomics Initiative [16] (MGI) at Michigan Medicine and the BioVU [15] at Vanderbilt University. In total, this consisted of 35,785,074 lab measurements in 50,743 MGI samples, and 28,929,660 lab measurements in 61,378 BioVU samples. We focused on samples of European ancestry in both cohorts due to insufficient sample sizes in other ancestry groups. Genetic analyses were performed on the set of ~800K overlapping SNPs between the MGI imputed genotypes and the BioVU MEGA array genotypes.

We analyzed 70 labs matched with high confidence between the health systems and having at least 1,000 samples with the lab measured in each health system (Table 1). We searched the GWAS catalog for known genetic associations among the 70 lab traits to serve as “true positive” variants to validate the data and assess competing analysis strategies (S2 Table). We identified 4,140 such associations, of which, 1,313 (32%) across 48 different traits were in the set of overlapping markers tested in the meta-analysis. Many lab traits have been well studied [28,29] and provided many testable catalog SNPs. LDL, for example, had 84 catalog SNPs that could be directly tested in our meta-analysis. Alternatively, several labs had relatively few or no catalog SNPs, including labs for which either no variant was reported in the catalog or the catalog variants were not typed in at least one of our cohorts.

**Table 1:** Summary of clinical lab traits tested, including meta-analysis samples size, number of testable GWAS catalog SNPs, number of replicated catalog SNPs and replication rate.

Lab Name	Category	Description	Meta-Analysis Sample Size	Number of Testable GWAS Catalog SNPs	Number of Catalog SNPs Replicated in Meta-Analysis	Replication Rate (%)
Alb	Liver function	Albumin, most abundant blood protein	39,513	5	4	80
AlkP	Liver function	Alkaline phosphatase, bile duct and bone enzyme released by damage	39,809	3	1	33
ALT	Liver function	ALanine aminoTransferase, liver enzyme released by damage	40,116	0	0	N/A
Amyl	Pancreas	Amylase, digestive pancreas enzyme released by damage	10,368	0	0	N/A
AST	Liver function	ASpartate aminoTransferase, liver enzyme released by damage	40,176	0	0	N/A
BasoAB	Differential	Basophils, white blood cell type (absolute number)	29,653	19	12	63
BasoRE	Differential	Basophils, white blood cell type (relative proportion)	32,578	11	7	64
BEAR	Blood gas	Base Excess ARterial, Acid-base measure of metabolic acidosis or alkalosis	8,895	0	0	N/A
Bili	Liver function	Total Bilirubin, heme byproduct excreted by liver	38,416	4	4	100
BNP	Heart failure	Brain Natriuretic Protein, Signaling protein from heart under stress	9,369	1	1	100
BUN	Renal function	Blood Urea Nitrogen Protein byproduct excreted by kidneys	45,922	0	0	N/A
Ca	Electrolytes	Calcium, blood electrolyte	46,100	9	7	78
Chol	Lipid panel	Total cholesterol	23,642	91	60	66
CKMBRe	Cardiac markers	Creatine Kinase Muscle Brain isoform, relative, Enzyme in heart released by damage	10,964	0	0	N/A
Cl	Electrolytes	Chloride, blood electrolye	45,920	0	0	N/A
CPK	Cardiac markers	Creatine PhosphoKinase, enzyme in skeletal and cardiac muscle released by damage	15,150	0	0	N/A
Creat	Renal function	Creatinine, creatine byproduct excreted by kidneys	46,027	36	29	81
CRP	Inflammatory	C-reactive protein, marker of inflammation	12,447	16	7	44
EoAB	Differential	Eosinophils, white blood cell type (absolute count)	29,912	31	25	81
EoRE	Differential	Eosinophils, white blood cell type (relative proportion)	26,980	28	18	64
Ferrit	Iron	Ferritin, iron storage protein	11,744	6	1	17
FT4	Thyroid function	Free tetraiodothyronin, active thyroid hormone	15,868	0	0	N/A
Gluc	Metabolic	Blood glucose	46,027	18	16	89
HCO3 (CO2)	Blood gas	Bicarbonate, main blood pH buffer	45,932	0	0	N/A
HCT	Complete blood count	Hematocrit, measure of blood oxygen carrying capacity	46382	36	20	56
HDL	Lipid panel	High density lipoprotein cholesterol	23,318	101	84	83
Hgb	Complete blood count	Hemoglobin, oxygen carrying protein	46,159	34	18	53
HgbA1C	Metabolic	Hemoglobin A1C, measure of blood glucose over previous 90 days	17,407	11	10	91
IGranAB	Differential	Immature granulocytes, immature white blood cell type (absolute count)	30,744	0	0	N/A
IGranRE	Differential	Immature granulocytes, immature white blood cell type (relative proportion)	30,683	0	0	N/A
INR	Coagulation	International Normalized Ratio, derivative of PT used to dose anticoagulants	33,695	0	0	N/A
Iron	Iron	Iron	11,317	4	3	75
K	Electrolytes	Potassium, blood electrolyte	45,941	0	0	N/A
LAC	Blood gas	Lactic acid, marker of tissue hypoxia	8,792	0	0	N/A
LDH	Tumor markers	Lactate dehydrogenase, enzyme found in many cell types released by damage	9,734	0	0	N/A
LDL	Lipid panel	Low density lipoprotein cholesterol	22,896	84	58	69
Lipase	Pancreas	Lipase, digestive pancreas enzyme released by damage	12,649	2	2	100
LymphAB	Differential	Lymphocytes, white blood cell type (absolute count)	32,548	35	22	63
LymphRE	Differential	Lymphocytes, white blood cell type (relative proportion)	32,553	20	10	50
MCH	Red cell indices	Mean corpuscular hemoglobin, used to differentiate causes of anemia	46,159	64	57	89
MCHC	Red cell indices	Mean corpuscular hemoglobin concentration, used to differentiate causes of anemia	46,157	20	19	95
MCV	Red cell indices	Mean corupuscular volume, used to differentiate causes of anemia	46,153	77	68	88
Mg	Electrolytes	Magnesium, blood electrolyte	22,773	4	4	100
MonoAB	Differential	Monocytes, white blood cell type (absolute count)	32,587	43	32	74
MonoRE	Differential	Monocytes, white blood cell type (relative proportion)	32,594	15	12	80
MPV	Coagulation	Mean platelet volume	40,058	84	73	87
Na	Electrolytes	Sodium, blood electrolyte	45,933	0	0	N/A
pCO2	Blood gas	Arterial partial pressure of CO2, measure of ventilation	9,516	0	0	N/A
pH	Blood gas	Arterial pH	10,279	0	0	N/A
Phos	Electrolyte	Phosphorus, blood electrolyte	21,618	5	4	80
PLT	Complete blood count	Platelet count, clot forming measure	46,145	102	84	82
PMNAB	Differential	Neutrophils, white blood cell type (absolute count)	32,595	35	15	43
PMNRE	Differential	Neutrophils, white blood cell type (relative proportion)	29,435	21	7	33
pO2	Blood gas	Arterial partial pressure of oxygen, measure of oxygenation	9,557	0	0	N/A
PT	Coagulation panel	Prothrombin time, clot forming measure	33,671	1	1	100
PTT	Coagulation panel	Partial Thromboplastin Time, clot forming measure	30,972	9	6	67
RBC	Complete blood count	Red Blood Cell count, measure of blood oxygen carrying capacity	46,158	50	31	62
RDW	Red cell indices	Red cell Distribution Width, measure of variability in MCV, used to differentiate causes of anemia	44,281	29	21	72
%SAT	Iron	Transferrin saturation, measure of available iron transport capacity	10,180	4	3	75
SedRat	Inflammatory markers	Erythrocyte Sedimentation Rate (ESR), non-specific marker of inflammation	13,945	5	5	100
TIBC	Iron	Total Iron Binding Capacity, measure of iron transport capacity, used to calculate transferrin saturation	10,397	1	1	100
TProt	Liver function	Total Protein in blood	38,352	2	2	100
Trigs	Lipid panel	Triglycerides, tested as part of cholesterol panels	23,963	73	63	86
Troponin	Cardiac markers	Troponin I, heart protein released by damage	10,106	0	0	N/A
TSH	Thyroid function	Thyroid Stimulating Hormone, test of thyroid function and feedback	27,441	1	1	100
UCrea	Renal function	Urine creatinine, measure of kidney function	10,522	0	0	N/A
UricA	Gout	Uric acid, nucleotide breakdown product elevated in gout	7,429	17	14	82
Vi-B12	Nutrition	Vitamin B12, used in DNA synthesis	12,506	7	7	100
Vit-D	Nutrition	Vitamin D storage form, regulates calcium and phosphorus	12,250	6	6	100
WBC	Complete blood count	White Blood Cell count	46,100	33	27	82
**TOTAL**				**1313**	**982**	**74.8**

### Meta-analysis of Labs in MGI and BioVU

**Fig 2:**
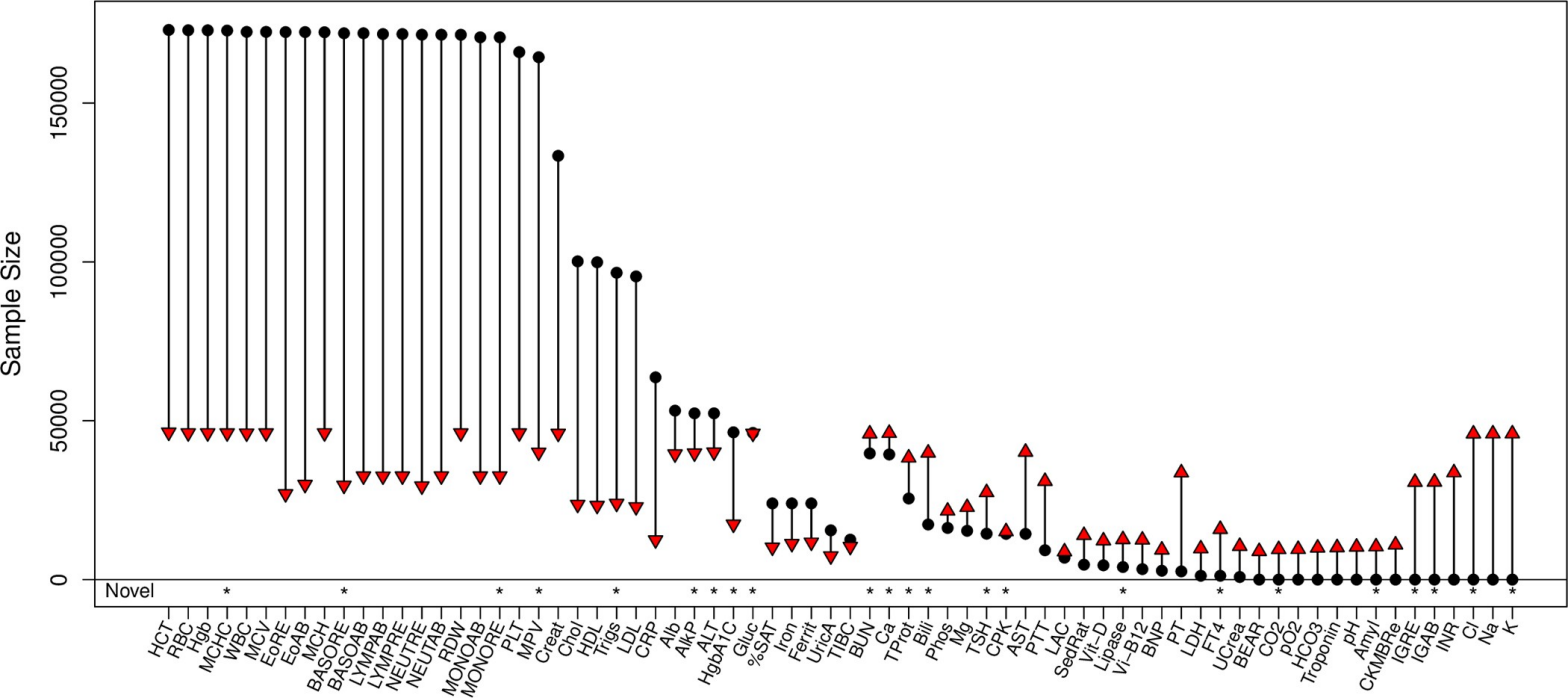
Sample sizes for 70 clinical lab traits from the meta-analysis of BioVU and MGI EHRs (red triangles) and the previous largest reported GWAS in a European cohort (black circles). Our meta-analysis provides the largest GWAS for 34 lab traits, including the first for 14. Asterisks along the bottom row indicate labs for which we identified a novel genetic association.

The 70 EHR-derived lab traits were first analyzed separately in the cohorts using the mean measurement as the individual-level outcome. The meta-analysis sample size differed between labs, ranging from 7,429 for uric acid to 46,382 for hematocrit (Fig 2), reflecting the frequency with which different labs are administered in health systems. Several labs have previously been studied in much larger cohorts, including the differential panel of 10 white blood cell measures, analyzed in >170K samples in the UK BioBank [29]. However, this meta-analysis provides the largest sample size for 34 labs, including 14 clinical lab traits with no previously reported study in the GWAS catalog at the time of our analysis. Genomic control lambda values (*λ*_GC_) confirmed the meta-analyses were well-controlled [30]. The mean *λ*_GC_ across all labs was 1.035, ranging between 0.995 and 1.103. Consistent with polygenicity [31], traits with a larger numbers of catalog variants had, on average, larger *λ*_GC_ values. The mean *λ*_GC_ for labs with zero testable catalog SNPs was 1.020. Labs with one to twenty testable Catalog SNPs had mean *λ*_GC_ of 1.028 and labs with greater than 20 testable Catalog SNPs had mean *λ*_GC_ of 1.066.

#### Replication of GWAS Catalog SNPs

We first performed a replication analysis of the 1,313 GWAS catalog SNPs to validate the EHR-derived lab traits. Overall, we replicated 982 of the GWAS catalog SNPs, giving an overall replication rate of 74.8% (Table 1). Replication rates varied across the individual labs; however, we did replicate at least one catalog SNP for each of the 48 traits with a testable catalog SNP. Replication rates were high for several previously well-studied traits, including red blood cell indices (MCHC, MCH, MCV), metabolic measures (glucose and HgbA1C) and creatinine. The lowest replication rates occurred for the differential panel of white blood cell traits (neutrophils, lymphocytes) which included catalog SNPs discovered in the much larger UK Biobank cohort [4]. Interestingly, replication rates differed among the well-studied lipid panel traits. We replicated a lower percentage of catalog SNPs for LDL cholesterol and total cholesterol compared to triglycerides and HDL cholesterol.

**Fig 3:**
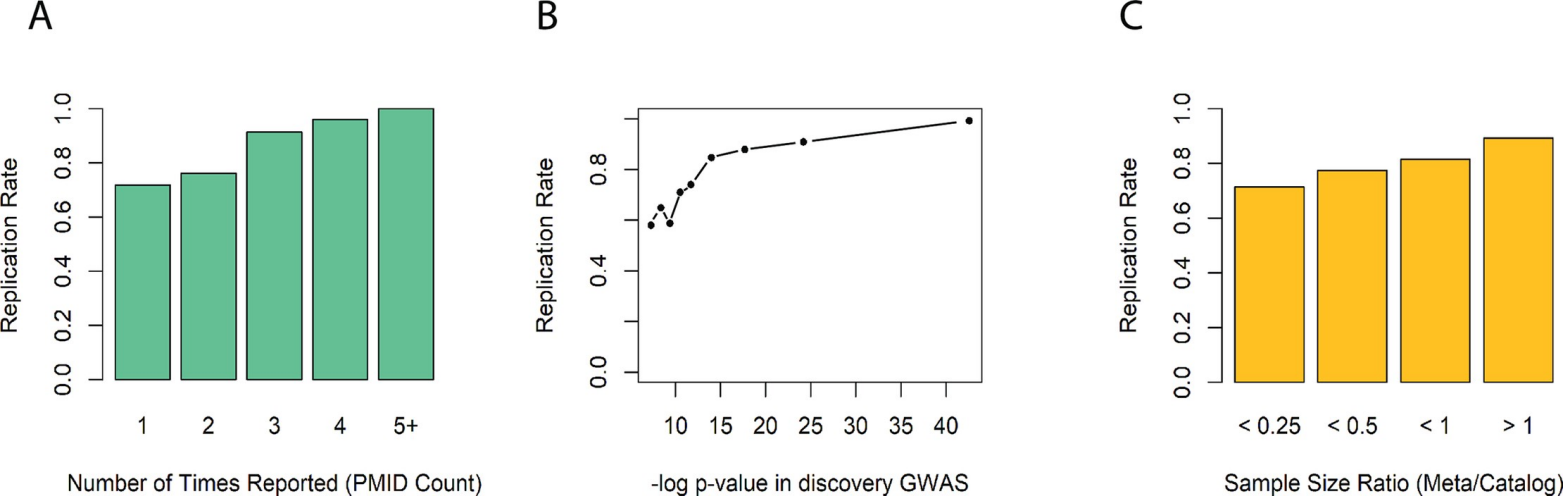
Replication rates for GWAS catalog SNPs of clinical labs increased with (A) the number of times an association was reported in the GWAS catalog, (B) the most significant p-value previously reported for the association, and (C) the ratio of sample size in our meta-analysis to that of the previous largest study.

Several factors influenced our ability to replicate individual catalog SNPs (Fig 3), each consistent with statistical power rather than adequate matching of labs as the primary limiting factor. Replication increased sharply with the number of publications reporting the association, as quantified using the PMID citation count from the GWAS catalog (Fig 3A). Associations reported only once in the catalog are a mix of true unreplicated associations and false positives, whereas associations reported more than once have already been replicated and are likely real. We replicated 70% (699 of 1000) of associations reported only a single time. That rate increased to 77% (196 of 256) for associations reported twice, 91% for associations reported three times and nearly 100% (56 of 57) for associations reported four or more times. Importantly, this analysis provides the first replication for 699 previously reported quantitative lab trait associations, increasing the likelihood that these are true genotype-phenotype associations (S2 Table).

Replication rate was also dependent on both the best previously reported p-value for the association and the sample size of the study reporting the association (Fig 3B & 3C). Our replication rate was lowest, between 55%-65%, for associations whose best reported p-value was just above genome-wide significance of 5e-8 but increased sharply thereafter. We replicated ~85% of catalog SNPs with best reported p-value <1e-15 and over 90% of catalog SNPs with best p-value <1e-20. Replication rate increased with the relative size of our meta-analysis compared to the largest reported study. We replicated approximately 90% of catalog SNPs for which our meta-analysis was at least as large as prior studies reporting the association.

#### Novel associations

We identified 264 SNP-lab trait pairs representing potentially novel associations. Based on visual inspection, these SNPs corresponded to 31 distinct peaks for which we report the lead SNP having the strongest association signal at each peak (Table 2).

**Table 2:** Summary of Novel findings.

					MGI-BioVU Meta-Analysis			BioVU Replication Cohort			
Lab	SNP	Chr:Pos	Allele 1	Allele 2	N	Beta	P-Value	N	Beta	P-Value	Replicated
AlkP	rs3843738	17:43739194	A	G	39,809	0.04	2.51E-08	22,920	0.01	3.58E-01	No
AlkP	rs73004933	19:19675696	T	C	39,809	0.08	4.47E-09	22,730	0.05	7.14E-03	Yes
ALT	rs112574791	8:145730221	A	G	40,116	0.18	3.02E-08	23,007	0.15	5.80E-04	Yes
Amyl	rs1930212	1:104324819	A	G	10,368	-0.25	1.48E-45	3,573	-0.18	4.69E-09	Yes
Amyl	rs8051363	16:75255217	A	G	10,368	0.10	1.07E-10	3,564	0.09	4.51E-04	Yes
BasoRE	rs386785158	15:70744437	T	C	29,653	0.06	7.94E-13	16,191	0.04	2.10E-04	Yes
Bili	rs855791	22:37462936	A	G	39,890	0.04	2.34E-08	22,918	0.04	1.00E-05	Yes
BUN	rs10516957	4:95949206	T	C	45,922	-0.06	1.35E-08	25,245	0.01	6.11E-01	No
Ca	rs6727384	2:97400324	A	G	46,100	-0.04	5.13E-10	25,200	-0.05	2.06E-07	Yes
Ca	rs2839899	9:80350999	A	G	46,100	0.04	6.76E-09	25,194	0.03	9.47E-03	Yes
Cl	rs1030025	2:103105611	A	T	45,920	0.05	4.68E-10	25,204	0.02	9.16E-02	No
FT4	rs10122824	9:139109861	T	G	15,868	0.07	1.00E-09	9,721	0.07	7.28E-07	Yes
Glucose	rs7607980	2:165551201	T	C	46,027	-0.05	4.27E-09	25,312	-0.04	2.09E-03	Yes
Glucose	rs896854	8:95960511	T	C	46,027	-0.04	1.55E-09	25,311	0.01	3.64E-01	No
Glucose	rs9273364	6:32626302	T	G	46,027	0.05	2.63E-11	24,801	0.05	3.10E-06	Yes
HgbA1C	rs3130628	6:31609272	T	C	17,407	-0.08	1.23E-08	7,340	0.03	3.79E-02	No
HCO3 (CO2)	rs1799913	11:18047255	T	G	45,932	-0.04	5.89E-09	25,219	-0.04	7.82E-07	Yes
HCO3 (CO2)	rs77375846	2:103155075	T	C	45,932	-0.10	9.33E-25	25,217	-0.06	2.78E-05	Yes
IGranRE	rs13284665	9:131513370	A	G	30,683	0.22	6.61E-74	QC Fail	N/A	N/A	No
IGranAB	rs13284665	9:131513370	A	G	30,744	0.13	6.76E-35	QC Fail	N/A	N/A	No
K	rs10039139	5:137164863	T	G	45,941	0.07	8.32E-16	25,211	0.06	1.83E-06	Yes
Lipase	rs9377343	6:96512220	A	G	12,649	-0.10	4.79E-14	5,564	-0.08	3.60E-05	Yes
Lipase	rs8051363	16:75255217	A	G	12,649	0.13	2.00E-20	5,549	0.07	8.39E-04	Yes
MCHC	rs12352830	9:80041132	C	G	46,157	-0.04	4.37E-08	26,243	-0.04	5.77E-05	Yes
MonoRE	rs117358683	12:44145965	A	G	32,594	-0.23	2.69E-08	16,185	0.04	4.07E-01	No
MPV	rs11212635	11:108310702	A	T	40,058	0.04	9.55E-09	17,333	-0.01	3.68E-01	No
TProt	rs8022180	14:103263020	A	G	38,352	0.04	7.24E-10	19,665	0.03	2.63E-03	Yes
Trigs	rs6847598	4:76750356	T	C	23,963	-0.05	1.58E-08	12,526	-0.03	1.48E-02	Yes
TSH	rs12590163	14:105223525	T	C	27,441	-0.05	4.68E-08	17,042	-0.04	6.76E-04	Yes
TSH	rs310766	3:12233482	A	G	27,441	-0.06	1.66E-08	17,079	-0.05	1.42E-05	Yes
TSH	rs9275141	6:32651117	T	G	27,441	0.05	3.47E-09	17,054	0.04	8.64E-04	Yes

We performed a replication analysis of the 31 lead SNPs using an independent cohort of 29,043 BioVU patients that became available after the initiation of our primary analysis. One SNP potentially novel for both immature granulocytes measures failed QC filtering in the replication cohort and could not be tested for replication. In total, we replicated 22 of the 31 (71%) novel associations (Table 2). Among the 24 replicated novel SNPs are the first associations for amylase (Amyl) and bicarbonate (CO2). We identified and replicated additional associations for alanine aminotransferase (ALT), alkaline phosphate (AlkP), Relative count of basophils (BasoR), total bilirubin (Bili), calcium (Ca), creatinine phosphokinase (CPK), glucose (gluc), mean corpuscular hemoglobin concentration (MCHC), lipase, and thyroid stimulating hormone (TSH).

Several of our novel findings have biological or existing evidence that support the association. Three of the associations have recently been identified for the same lab in non-European cohorts. rs855791, a missense variant in *TMPRSS6* (transmembrane serine protease 6), and rs8022180, an intronic variant in *TRAF3*, were shown to be associated with bilirubin and serum total protein level, respectively, in a Japanese population [11]. rs112574791 is in the glutamic—pyruvic transaminase gene *GPT*, a gene associated with alanine aminotransferase levels in the Korea Biobank [32]. Our results confirm these prior findings and suggest a cross-ethnic effect in European populations.

The intronic variant rs8051363 in *CTRB1* was associated with both amylase and lipase, clinical assays of pancreas function used to diagnose pancreatitis. While the SNP itself has previously been linked to blood protein measurements [33], the *CTRB1* gene encodes chymotrypsin, a component of digestive enzyme secreted by the pancreas, and was previously shown to be associated with alcoholic chronic pancreatitis [34]. A second novel SNP for lipase, rs9377343, is an intronic variant in *FUT9*, a gene that showed association with diabetic neuropathy in a trans-ethnic meta-analysis [35].

The amylase-associated SNP rs1930212 resides near three amylase genes (*AMY2B*, *AMY2A* and *AMY1*) on chromosome 1, each of which encodes enzymes that digest starch into sugar [36]. Copy number variation for amylase genes is hypothesized to have been subject to selective sweeps corresponding to starch content in human diets [37]. The rs1930212 SNP tags a known deletion of *AMY2A*, a pancreatic amylase enzyme, most common in populations historically lacking starch rich diets [37].

One of our novel results for calcium, rs2839899, is an intronic variant in *GNAQ* (G protein subunit alpha q), a signaling protein involved in response to various hormones. Variation in *GNAQ* is associated with Sturge-Weber syndrome [38], a hereditary vascular malformation syndrome which can lead to deposits of calcium (calcification) in the brain.

Three SNPs showed associations with glucose. rs7607980 is a missense variant in *COBLL1* previously linked to fasting blood insulin and Type 2 diabetes [39–41]. rs9273364 is located near HLA-DQB1-AS1, a gene associated with T2D [42]. And, although it did not replicate in our analysis, rs896854, a variant mapping to both *NDUFAF6* and *TP53INP1*, has recent associations with T2D [43] and eosinophil count [44] among UK biobank participants.

We note that several associations occurred within the HLA region on chromosome 6, notably for glucose, hemoglobin A1C, and TSH. These variants are likely segregating with HLA types, which are strongly associated with various autoimmune diseases including diabetes and autoimmune thyroiditis, which have strong effects in these particular labs.

#### Genetic correlation of clinical labs

**Fig 4:**
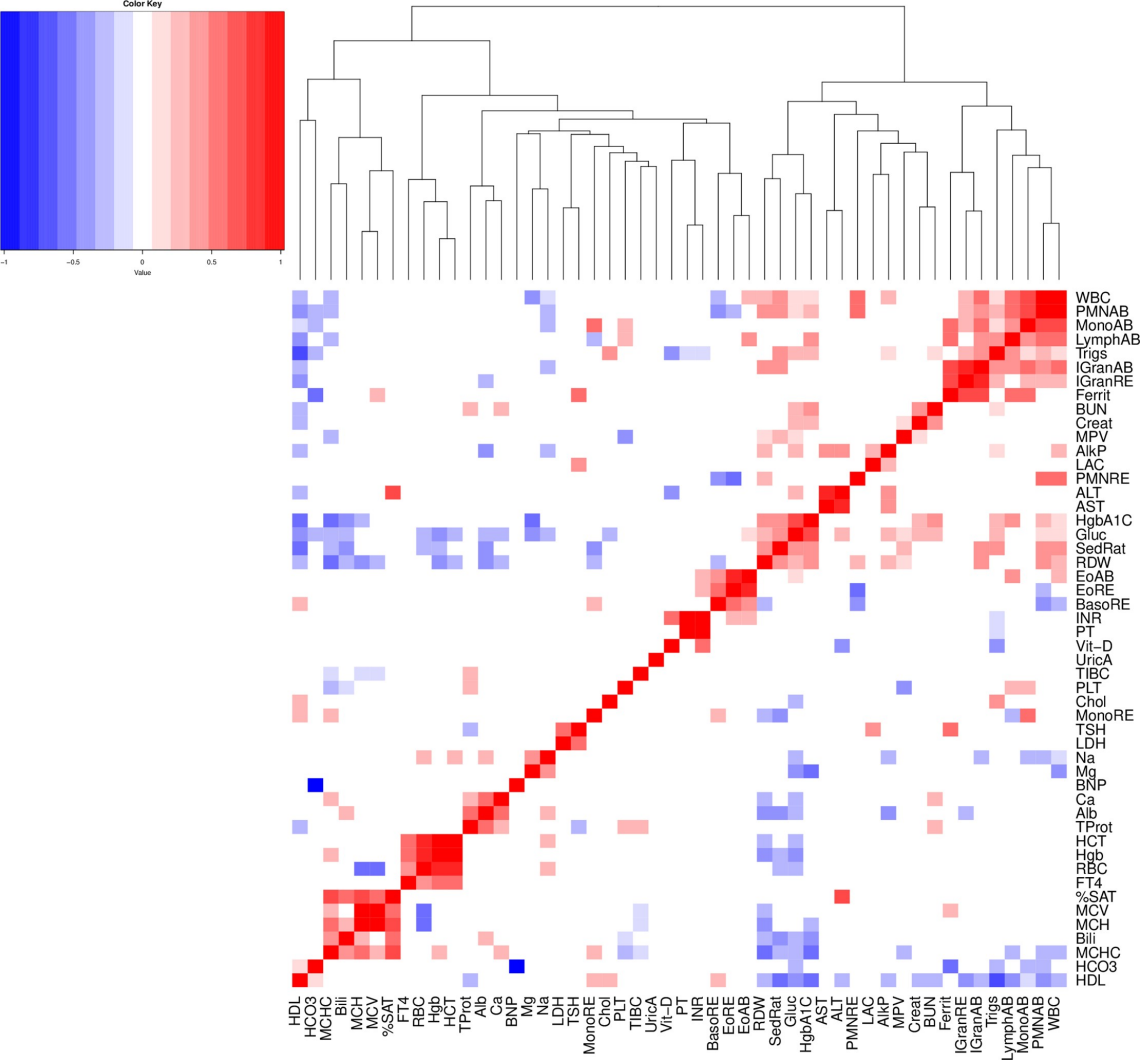
Pairwise genetic correlation of clinical lab traits. We restricted to labs with heritability of at least 7%. Squares are colored only for correlations having a p-value <0.05 for the null hypothesis of correlation equal to zero.

We computed the genetic correlation between pairs of labs using LD score regression [45] to learn about the shared genetic basis of these traits (Fig 4). We restricted analysis to the 50 lab traits with heritability of at least 7% based on recommendations by the developers of LDscore regression that estimation of genetic correlation can be unreliable when one of trait has heritability close to zero. We observe strong positive correlations among lab traits of similar function. For example, the liver enzymes alanine aminotransferase (ALT) and aspartate aminotransferase (AST) were strongly correlated, as were the measures of renal function Blood Urea Nitrogen (BUN) and creatinine (Creat). Prothrombin time (PT), a measure of clot formation time and a derivative measure International Normalized Ratio (INR) were positively correlated as expected. More surprisingly, INR was also positively correlated with vitamin D. While vitamin K is known to be required for the formation of prothrombin, the correlation with Vitamin D suggests a potential covariance in nutrition or nutrient absorption.

A prominent cluster of labs (top right corner of the heatmap) contains primarily white blood cell traits including measures of immature granulocytes, lymphocytes, monocytes and neutrophils. The immature granulocytes also showed a strong correlation with ferritin (ferrit), an iron storage and acute phase protein. Ferritin and immature granulocytes can both be elevated during severe acute inflammation, explaining this correlation.

As expected, HgbA1C and glucose were strongly correlated. More interestingly, they also clustered with Red cell Distribution Width (RDW) and Erythrocyte Sedimentation Rate (SedRat). This cluster of labs showed negative associations with high density lipoprotein (HDL), mean cell hemoglobin concentration (MCHC), and mean cell hemoglobin (MCH). This supports a pathophysiology where the metabolic syndrome (obesity, elevated glucose, low HDL) is linked by complex mechanisms to persistent low-level inflammation (elevated SedRat), and anemia of chronic disease (elevated RDW, low MCH, low MCHC).

We identified a cluster containing red cell indices–mean cell hemoglobin concentration (MCHC), mean cell hemoglobin (MCH), and mean cell volume (MCV)–with total bilirubin (Bili) and transferrin saturation (%SAT). This reflects the biology of hemoglobin–iron is carried to red cell precursors by transferrin and incorporated into heme and thence hemoglobin, red cells are filled with hemoglobin, and at the end of a red cell lifecycle, heme is broken down into bilirubin.

Additional clusters include (1) calcium (Ca), albumin (Alb) and total protein in blood (TProt), (2) thyroid stimulating hormone (TSH) and lactate dehydrogenase (LDH), and (3) hematocrit (HCT), red blood cell count (RBC) and hemoglobin (Hgb) with free tetraiodothyronine (FT4). Albumin (Alb) is the major blood protein, so Alb levels are unsurprisingly correlated with total blood protein (TProt). Calcium homeostasis is driven by free calcium, while albumin acts as a calcium sink, therefore calcium (Ca) levels would reasonably be expected to correlate with Alb [46].

Hematocrit (HCT), red blood cell count (RBC) and hemoglobin (Hgb) are interrelated measures of oxygen carrying capacity in blood and unsurprisingly correlated. In our study, they are also correlated with free tetraiodothyronine (FT4). Anemia (low HCT, RBC, and Hgb) may be a feature of hypothyroidism (low FT4), and tetraiodothyronine—thyroid hormone—has been reported to play a role in red cell maturation [47,48].

A final cluster was identified linking thyroid stimulating hormone (TSH) to lactate dehydrogenase (LDH). Muscle breakdown, manifesting as weakness, is a feature of hypothyroidism, and therefore other laboratory anomalies seen in hypothyroidism include release of muscle enzymes including LDH [47,49].

### Analytic strategies for EHR-derived lab traits

We explored the impact of analytic choices on downstream analysis by performing parallel GWAS analyses in the MGI and BioVU cohorts with one of the analytic steps perturbed from our original analysis: either the individual-level statistic used to summarize longitudinal lab measurements (median, maximum measurement, first available measurement) or the inclusion of covariates for underlying comorbid health conditions. We performed these analyses on the 22 lab traits for which there were least 20 testable GWAS catalog SNPs, using the catalog SNPs to interpret the effect of each analytic strategy on true risk variants.

#### Summary statistic

Overall, 13.3% of testable catalog SNPs showed a major change in significance when using the median as opposed to mean value for the summary statistic (Table 3). The median rarely resulted in a consistent improvement for both MGI and BioVU. Only 0.4% of catalog SNPs had concordant increased effect compared to 7.6% with concordant decreasing effect and 5.2% with a discordant effect. Creatinine was the sole lab for which using median lab value had a greater number of catalog SNPs with concordant increased significance than catalog SNPs with concordant decreased significance. Even here the effect was small, only two of the 36 catalog SNPs had a concordant increase in significance.

**Table 3:** Classification of catalog SNPs for alternate summary statistics.

		Median Measurement			First Available Measurement			Maximum Measurement		
Lab	Testable Catalog SNPs	Concordant Increased Significance	Concordant Decreased Significance	Discordant Effect	Concordant Increased Significance	Concordant Decreased Significance	Discordant Effect	Concordant Increased Significance	Concordant Decreased Significance	Discordant Effect
Chol	91	0	12	0	1	11	1	2	4	6
Create	36	2	0	0	2	2	1	0	8	1
EoAB	31	0	6	0	0	9	0	0	2	1
EoRE	28	0	1	0	0	4	0	0	1	1
HCT	36	0	0	0	4	0	1	15	0	1
HDL	101	0	6	3	0	15	1	0	27	5
Hgb	34	0	0	0	5	0	0	12	0	0
LDL	84	0	9	1	0	9	4	2	2	6
LymphAB	35	0	0	0	0	3	1	5	1	2
LymphRE	20	0	0	0	0	0	0	0	0	0
MCHC	20	0	1	0	2	5	3	2	5	1
MCH	64	0	16	27	0	33	8	0	33	7
MCV	77	1	5	7	0	19	13	0	30	6
MonoAB	43	2	3	0	0	9	0	0	13	1
MPV	84	0	11	9	0	39	9	5	20	17
PLT	102	0	0	1	7	7	1	0	19	5
PMNAB	35	0	0	0	0	2	1	0	3	1
PMNRE	21	0	0	0	0	0	0	0	0	1
RBC	50	0	4	4	13	0	1	21	0	0
RDW	29	0	1	2	0	1	4	0	7	0
Trigs	73	0	7	0	1	15	1	0	22	0
WBC	33	0	4	5	0	7	1	0	9	0
Total	1127	5 (0.4%)	86 (7.6%)	59 (5.2%)	35 (3.1%)	190 (16.9%)	51 (4.5%)	64 (5.6%)	206 (18.3%)	62 (5.5%)

In comparison, the first available measurement and the maximum measurement had a greater impact on association p-values for catalog SNPs. In both cases, the alternate summary statistic was most likely to cause a concordant decrease in significance. Using the first available measurement resulted in concordant increase for only 3.1% of catalog SNPs, whereas 16.9% of catalog SNPs had a concordant decrease and 4.5% had discordant changes in significance. Using the maximum available measure had similar performance (5.6% concordant increase, 18.3% concordant decrease, 5.5% discordant).

Despite an overall trend of reducing significance of known risk variants, several related labs for blood oxygen carrying capacity did benefit from using the first available or maximum measurements. Red blood cell count (RBC), hematocrit (HCT) and hemoglobin (Hgb) each showed concordant increase in significance for several of their respective catalog SNPs without negatively impacting remaining catalog SNPs. This likely reflects red cell biology. Conditions that decrease oxygen carrying capacity, such as blood loss or iron deficiency are far more common than those that increase it, polycythemia vera or severe obstructive sleep apnea, for example. Thus, maximum measurement of an individual’s oxygen carrying capacity more likely represents the genetically determined set point.

#### Controlling for comorbid disease

The comorbidity model, containing binary covariates for 42 comorbid diseases with the potential to alter lab values, produced the largest proportion of catalog SNPs (6.2%) with concordant increased significance in MGI and BioVU among the alternate analysis strategies considered (Table 4). Despite this, a roughly equal number of catalog SNPs had discordant effects (6.8%) between the two cohorts.

**Table 4:** Classification of catalog SNPs for the comorbidity model, which includes covariates for various lab-altering diseases.

		Comorbidity Model		
Lab	Testable Catalog SNPs	Concordant Increased Significance	Concordant Decreased Significance	Discordant Effect
Chol	91	2	5	2
Creat	36	1	3	2
EoAB	31	0	0	0
EoRE	28	0	0	1
HCT	36	2	0	2
HDL	101	15	2	2
Hgb	34	1	0	0
LDL	84	0	7	2
LymphAB	35	2	0	4
LymphRE	20	0	0	0
MCHC	20	2	0	2
MCH	64	1	7	26
MCV	77	9	1	4
MonoAB	43	5	0	1
MPV	84	18	0	5
PLT	102	5	1	4
PMNAB	35	0	2	1
PMNRE	21	0	0	2
RBC	50	2	0	5
RDW	29	0	1	3
Trigs	73	3	3	7
WBC	33	2	2	2
Total	1127	70 (6.2%)	34 (3.0%)	77 (6.8%)

The clearest example of a substantial and consistent effect on catalog SNPs between MGI and BioVU was for HDL and Mean platelet volume (MPV). Interestingly, in contrast to this result for HDL, LDL had no catalog SNPs with concordant increase in significance and seven catalog SNPs with concordant decrease.

## Discussion

This study represents the first cross-health system study of EHR-derived lab traits at large scale. We performed meta-analysis GWAS of 70 lab traits and have made these association results easily accessible to the research community. Thoroughly dissecting each lab-SNP combination is a daunting task. Here, we focused on replication of GWAS catalog variants to validate our data and highlighted novel genetic associations. We anticipate that our full results, including those which do not reach genome-wide significance will be useful in replicating future novel results, in studies which synthesize findings across multiple SNPs, or in hypothesis-driven studies which require less stringent thresholds.

Our study serves as a proof-of-principle for performing cross-health-system genetic analysis of EHR-derived lab traits. The high replication rate for known GWAS variants indicates that EHR lab traits can be well-matched between discordant health systems and that measurements taken during real-life medical interactions sufficiently reflect those taken under more idealized experimental conditions. Moreover, this implies that mechanisms underlying variation in lab traits among healthy populations also act in a health system population with diseased individuals, strengthening their clinical relevance. By comparing various analytic strategies, we show that there is no optimal strategy that holds across all lab traits. In fact, we observed many instances in which the alternate analysis simultaneously increased significance for some risk variants and decreased significance for others. Thus, even within a given lab trait, an optimal strategy for variant discovery might not exist. We also considered a summary statistic based on Area Under the Curve for the longitudinal lab data [50,51]. Analysis in the MGI cohort showed that this measure performed consistently worse than the mean lab measurement (S1 Text, S3 Table). A potential area of future research would be determining if multiple versions of a lab trait can be combined into an omnibus test that simultaneously increases power across all risk variants. We encourage researchers to use our results across the various analysis strategies to guide decisions about how best to analyze their traits of interest.

The primary strength of our study was the access to two independent biobank cohorts. Using two cohorts provides an increase in sample size and power over analyzing and reporting on each cohort separately. In addition, the two-cohort design adds a built-in internal consistency check to our results by requiring effect sizes to be in the same direction in both cohorts. This additional requirement reduced the potential for unknown biases in the health system cohorts to create spurious results when replicating GWAS catalog SNPs or novel association discovery. Further, the independent cohorts provided the means to rigorously examine the portability of analytic strategies between biobanks. A similar analysis performed in a single cohort could produce recommendations over fitted to one specific context. Use of multiple sites increases the generalizability of our recommendations. This study was further strengthened by the fortuitous availability of an independent tranche of BioVU samples that provided an immediate replication cohort for the novel findings of our meta-analysis.

Our study has implications for the design and analysis of similar studies in the future. Matching and analyzing lab data between health systems is difficult and requires substantial content knowledge. This study benefited from a multi-disciplinary team consisting of clinical experts to lead the categorization of the raw lab data extracts and statistical geneticists to guide analytic strategies. We leaned heavily on GWAS catalog SNPs to serve as positive controls. We recommend researchers to incorporate an explicit replication step to validate lab data prior to testing novel hypotheses. Summarizing the longitudinal measurements simply using the mean proved relatively robust across labs but was by no means optimal in all scenarios. Future studies can benefit from considering a summary statistic suited to the specific lab trait being evaluated. Our analysis also highlights that close attention must be paid to differences in the preparation and analysis of EHR phenotypes, particularly longitudinal lab measurements. Failing to replicate a prior finding can be due to lack of a true effect but also a variety of differences between biobank cohorts and analytic procedures.

We were motivated to examine the effect of controlling for disease status because of its use in the analysis of lab traits in BioBank Japan [11]. Controlling for diseases or risk factors such as tobacco use is a common practice [29]. We considered testing the effect of each disease individually but discarded it as cumbersome. Our strategy reflects a broad-spectrum approach in which diagnoses that are rare or have limited effect on a lab can be rationalized as not causing harm by remaining in the model. The effect of controlling for comorbid diseases can be unpredictable. For example, within the components of a lipid panel, controlling for disease status led to a net improvement for HDL catalog SNPs, a net worsening for LDL catalog SNPs, and had cohort-specific impact on triglycerides. From a methodological standpoint, this argues for careful consideration of comorbid disease covariates. From a practical standpoint, the absence of diagnostic data should not be seen as precluding use of a clinical lab data.

A limitation of studying clinical labs in real-life cohorts is that some measurements will be affected by medication. We were unable to formally address the effect of medication because of unreliable measurements of medication. However, it remains an important consideration for future EHR-based lab studies and requires further study. There was indication that in situations where a disease diagnosis is likely to be accompanied by medication, for example a diagnosis of dyslipidemia with lipid labs, controlling for disease status diagnosis serves as a reasonable proxy to treatment status. As research interest in EHR phenotypes increases, we anticipate that improved capture of prescription data will facilitate the identification of medication effects.

A further limitation of this study is the number of analyzed genetic variants. The study was restricted to ~800K SNPs because BioVU imputed genotypes were unavailable at time of analysis. Although this limited our ability to discover novel variation, the number of SNPs was more than sufficient to perform the primary purpose of the paper, a proof-of-principle replication analysis across a broad range of clinical labs and analytic strategies. However, there are likely many loci remaining to be discovered for these labs, particularly the understudied traits.

In conclusion, we report the first lab-wide genome-wide association study linking data between two independent EHR-based cohorts. We achieved a high degree of replication of prior associations and report a modest number of new associations. In melding these data sets, we addressed key questions in design and analysis of ‘real world’ data that are increasingly relevant.

## Supporting information

S1 Table List of ICD-10 codes used for defining binary trait comorbidities in MGI and BioVU participants for the comorbidity GWAS model. (XLSX)

S2 Table Table of 1,313 SNPs extracted from the GWAS Catalog based on prior associations with the lab traits and SNPs considered in this study. These associations have been reported at least once in a mixed-sex, adult, European-predominant population not selected for the presence of any disease.
(XLSX)

S3 Table Comparison of GWAS results based on the Area Under the Curve (AUC) summary statistic and the default mean value summary statistic. (PDF)

S1 Text Methodological description of the GWAS analysis of lab traits using a summary statistic based on Area Under the Curve (AUC). (PDF)

S1 Fig The following set of scatterplots show the -log_10_ fold changes in p-value at individual SNPs when comparing GWAS of our default summary statistic (mean) to GWAS based on an alternative statistic (median, maximum or first available). Please refer to the Methods section for a complete description. The x-axis corresponds the fold changes for the SNP in MGI and the y-axis corresponds to the fold changes for BioVU. Positive log-fold changes indicate that the alternative statistic yielded a smaller (more significant) p-value than using the mean as a summary statistic. The upper-right (green) quadrant plots SNPs that decreased in p-value in both cohorts for the alternative statistic. The lower-left (blue) quadrant plots SNPs that increased in p-value in both cohorts. The two remaining quadrants indicate SNPs with discordant changes in p-value between the cohorts. GWAS catalog SNPs are plotted in red, novel SNPs for a given lab (if applicable) are plotted in purple, and the remaining SNPs are LD-pruned (for plotting convenience) and plotted in black. The white diamond displays an empirical null distribution of fold changes for non-associated SNPs. The first 22 pages display the three alternative summary statistics (maximum value, median value, and first available measurement) for a single lab. The following six pages contain the analogous plots showing log fold change in p-values for the comorbidity model, which includes binary covariates for various comorbid diseases with the potential to impact lab measures, to a default analysis that does not account for comorbidities.
(PDF)

S2 Fig (PDF)
