# Supplementary material for: LabWAS: Novel findings and study design recommendations from a meta-analysis of clinical labs in two independent biobanks
Source: PLoS Genet. 2020 Nov 11;16(11):e1009077. doi: 10.1371/journal.pgen.1009077 (PMC7682892; doi:10.1371/journal.pgen.1009077)
Supplement: S3 Table — (PDF) [file pgen.1009077.s003.pdf]

Lab	Normal Range	Units	Number of Catalog SNPs	Smaller p-value for AUC	Larger p-value for AUC	χ^2 test for p-value change	Median fold change
Chol	(-Inf, 200)	mg/dL	92	20%	80%	5.3E-09	5.5
Creat	(0.7, 1.3)	MG/DL	36	25%	75%	2.7E-03	11.1
EoAB	(-Inf, 0.8)	K/MM3	31	35%	65%	1.1E-01	14.9
EoRE	(-Inf, 6)	%	28	36%	64%	1.3E-01	4.4
HCT	(39, 50.2)	%	36	28%	72%	7.7E-03	12.4
HDL	(40, Inf)	mg/dL	102	13%	87%	5.3E-14	44.1
Hgb	(13.5, 17)	g/dL	34	32%	68%	4.0E-02	2.2
LDL	(-Inf, 100)	mg/dL	85	19%	81%	9.0E-09	8.1
LymphAB	(0.8, 5)	K/MM3	35	26%	74%	4.1E-03	9.3
LymphRE	(20.5, 45.5)	%	20	35%	65%	1.8E-01	4.5
MCH	(27, 32)	pg	64	6%	94%	2.6E-12	5744.8
MCHC	(32, 36)	g/dL	20	10%	90%	3.5E-04	1119.8
MCV	(81, 99)	fl	77	9%	91%	7.0E-13	2916.6
MonoAB	(0.1, 1)	K/MM3	43	12%	88%	4.8E-07	258.4
MPV	(9, 12.2)	fl	84	6%	94%	6.8E-16	13873.8
PLT	(150, 400)	K/MM3	102	10%	90%	4.7E-16	37.4
PMNAB	(1.8, 10.1)	K/MM3	35	29%	71%	1.1E-02	2.1
PMNRE	(43, 65)	%	21	33%	67%	1.3E-01	1.3
RBC	(3.9, 5.3)	M/MM3	50	20%	80%	2.2E-05	5.8
RDW	(11.5, 15)	%	29	14%	86%	9.6E-05	13.2
Trigs	(-Inf, 150)	mg/dL	74	15%	85%	1.5E-09	77.9
WBC	(4, 10)	K/MM3	33	15%	85%	6.2E-05	9.7
