# Supplementary material for: LabWAS: Novel findings and study design recommendations from a meta-analysis of clinical labs in two independent biobanks
Source: PLoS Genet. 2020 Nov 11;16(11):e1009077. doi: 10.1371/journal.pgen.1009077 (PMC7682892; doi:10.1371/journal.pgen.1009077)
Supplement: S1 Text — (PDF) [file pgen.1009077.s004.pdf]

We performed a GWAS of lab traits in MGI samples using an Area Under the Curve (AUC) approach to summarize the longitudinal lab measurements. We performed the analysis on the 22 lab traits with 20+ catalog SNPs and compared performance to GWAS with arithmetic mean as the outcome. To construct the AUC statistic, we defined clinically relevant thresholds for each lab trait to differentiate normal variation in measurements from measurements potentially indicative of underlying disease (Table S2, below). The thresholds were based on Normal Range criteria in the EHR and clinical guidance of co-authors. A lab trait could have either an upper threshold, a lower threshold or both depending on the clinical use for diagnosis. For example, the normal range for Red Blood Cell Count (RBC) trait had a lower threshold of 3.9 M/MM3 and an upper threshold of 5.3 M/MM3 since both low and high RBC measurements can be indicative of health problems. In contrast, Low-Density Lipoprotein (LDL) had only an upper threshold of 100 mg/dL since high values of LDL are clinically relevant for disease diagnosis.

We computed individual-level summary statistics that account for both the magnitude and duration of time that longitudinal lab measurements were outside the normal range thresholds.

For a given lab trait, let t_l be the lower threshold and t_u be the upper threshold for clinical relevance. Let m be the jth lab measurement in the ith subject. Any measurement satisfying $t_l \leq m \leq t_u$ is therefore within the "Normal" range of measurements for the given lab trait. Next, define

$$y_{ij} = \begin{cases} m_{ij} - t_u & \text{if } m_{ij} > t_u \\ 0 & \text{if } t_l \leq m_{ij} \leq t_u \\ m_{ij} - t_l & \text{if } m_{ij} < t_l \end{cases}.$$

The quantity y_{ij} is therefore equal to zero if the measurement m_{ij} is in the Normal range and equal to the amount outside the Normal range otherwise. Notably, y_{ij} is positive if the measurement m_{ij} is above the upper threshold and negative if it is below the lower threshold. Thus measurements above the upper threshold accumulate positive area and measurements below the lower threshold accumulate negative area. We set $t_l = -\infty$ for labs with only an upper threshold and $t_u = \infty$ for labs with only a lower threshold.

We computed the accumulated Area Under the Curve statistic for the ith sample based on the Trapezoidal Method as follows:

$$AUC_i = \sum_{j=1}^{n_i - 1} \frac{1}{2} \Delta_{ij} \left(y_{ij} + y_{ij+1}\right),$$

where n_i is the number of measurements for the ith sample and Δ_{ij} is the time (in days) between the jth and $(j+1)^{st}$ measurements for the ith sample. For an individual with only a single measurement, we defined the AUC as equivalent to the value of y_{i1}. We performed GWAS of the AUC values using the same procedure described in the Methods section of the main text.

We found that the AUC-based GWAS performed poorly compared to the standard GWAS of mean trait value based on change in p-values for GWAS catalog SNPs (Table S2). For each lab trait, we computed the proportion of catalog SNPS with smaller p-values (increased significance) and larger p-values (decreased significance) in the AUC GWAS. Assuming the AUC statistic and the mean statistic are equally

powerful for summarizing the longitudinal lab measures, we expect that p-values for AUC GWAS will increase for 50% of catalog SNPs on average and decrease for approximately 50% solely due to chance. We found that for all 22 lab traits tested, most catalog SNPs had larger p-values for the analysis based on the AUC summary statistic. That is, the AUC statistic resulted in p-values that with reduced significance. The imbalance was quite extreme: 9 labs traits had >80% of catalog SNPs increase in magnitude and 18 lab traits had p<0.05 for a χ^2 test for equal proportions of catalog SNPs with increasing and decreasing significance. Further, we computed the median fold change across all catalog SNP p-values for each, where fold change greater than 1 indicates a larger p-value for the AUC analysis. All 22 lab traits have median fold change >1 further emphasizing the overall reduced performance of our AUC statistic compared to the standard mean statistic.

The AUC style statistic presents several attractive features for summarizing complex longitudinal lab data; however, we found our implementation to be poor in comparison the basic mean value. We suggest some key limitations of the AUC statistic when applied to the lab measurements. First, restricting the AUC to area outside the clinically thresholds is akin to a censoring natural trait variation and reducing the effective sample size to patients with non-Normal measurements. As an example, 14.5% of samples in the LDL GWAS had AUC values of 0 because none of the LDL measurements for these sample were above the clinical threshold. The use of clinical thresholds is further complicated by the potentially subjective nature of their selection. Second, the AUC statistic can be unduly influenced by individual outlier measurements, particularly those that occur far in time from other measurements. Because the AUC statistic accounts for time between measurements through the Δ_{ij} term, a single measurement outside clinical thresholds is dramatically upweighted if no other measures are taken closely in time afterward. This property is particularly problematic because EHR lab measurements represent a highly imbalanced study design in which successive measurements can routinely occur far apart in time. The AUC statistic might be more effective in a balanced study design with similar numbers of lab measurements at fixed time intervals.

Despite the poor performance here, further refinement in the implementation of an AUC-style summary statistic for EHR lab data could produce more favorable results and is an area for future research.
