# Supplementary material for: LabWAS: Novel findings and study design recommendations from a meta-analysis of clinical labs in two independent biobanks
Source: PLoS Genet. 2020 Nov 11;16(11):e1009077. doi: 10.1371/journal.pgen.1009077 (PMC7682892; doi:10.1371/journal.pgen.1009077)
Supplement: S1 Fig — Please refer to the Methods section for a complete description. The x-axis corresponds the fold changes for the SNP in MGI and the y-axis corresponds to the fold changes for BioVU. Positive log-fold changes indicate that the alternative statistic yielded a smaller (more significant) p-value than using the mean as a summary statistic. The upper-right (green) quadrant plots SNPs that decreased in p-value in both cohorts for the alternative statistic. The lower-left (blue) quadrant plots SNPs that increased in p-value in both cohorts. The two remaining quadrants indicate SNPs with discordant changes in p-value between the cohorts. GWAS catalog SNPs are plotted in red, novel SNPs for a given lab (if applicable) are plotted in purple, and the remaining SNPs are LD-pruned (for plotting convenience) and plotted in black. The white diamond displays an empirical null distribution of fold changes for non-associated SNPs. The first 22 pages display the three alternative summary statistics (maximum value, median value, and first available measurement) for a single lab. The following six pages contain the analogous plots showing log fold change in p-values for the comorbidity model, which includes binary covariates for various comorbid diseases with the potential to impact lab measures, to a default analysis that does not account for comorbidities. (PDF) [file pgen.1009077.s005.pdf]

Chol, first

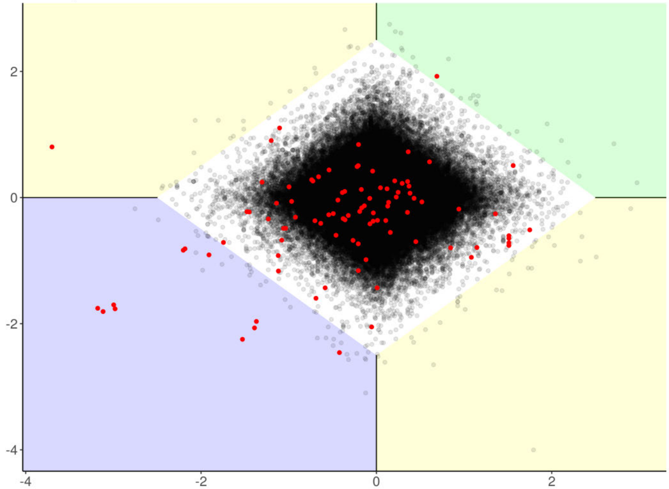

Chol, max

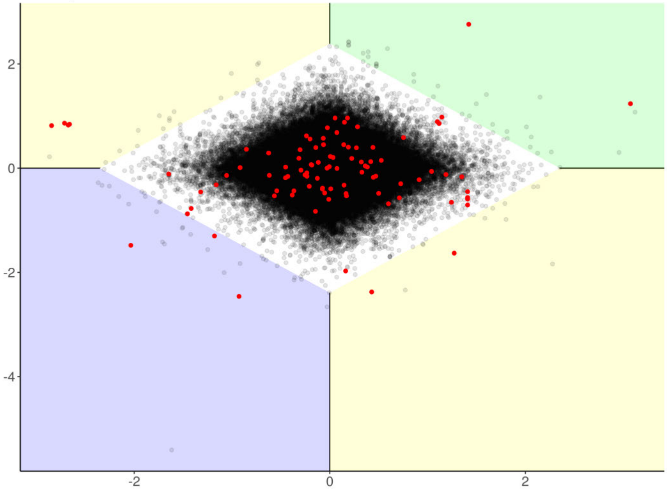

Chol, median

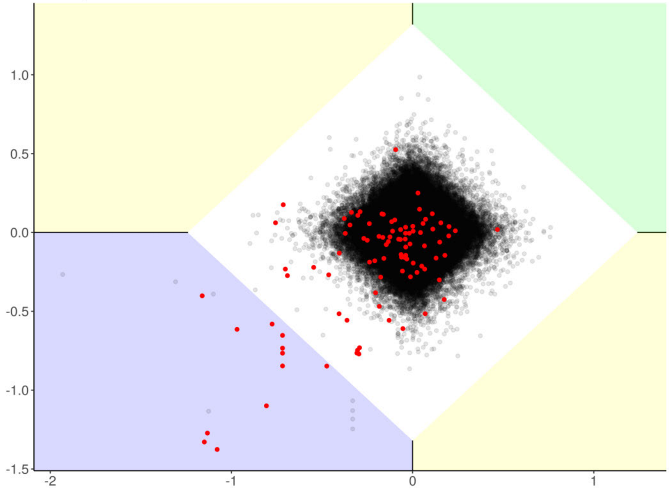

Creat, first

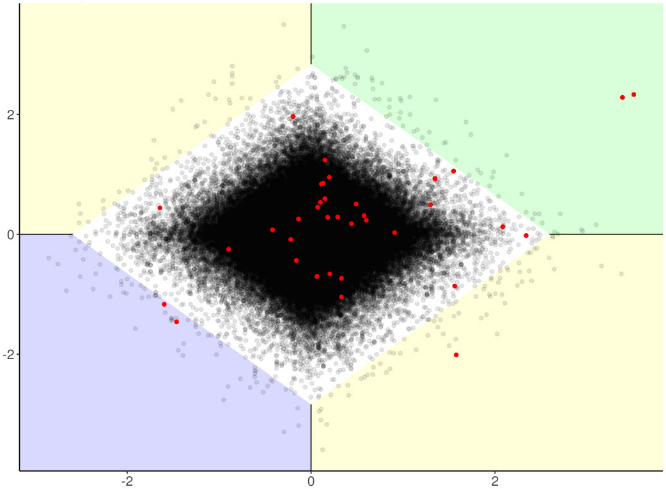

Creat, max

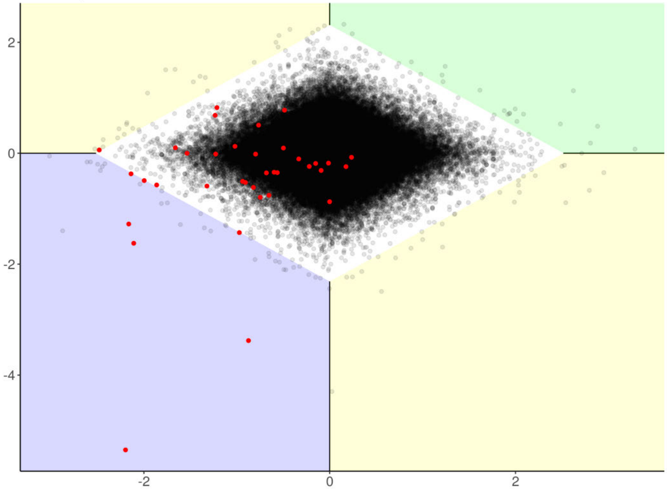

Creat, median

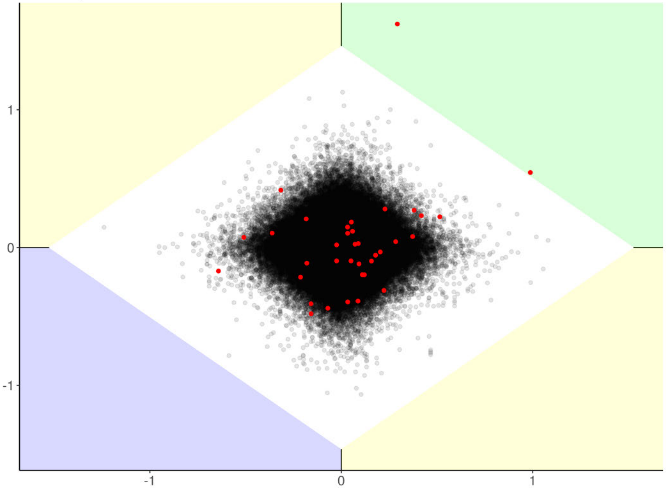

EoAB, first

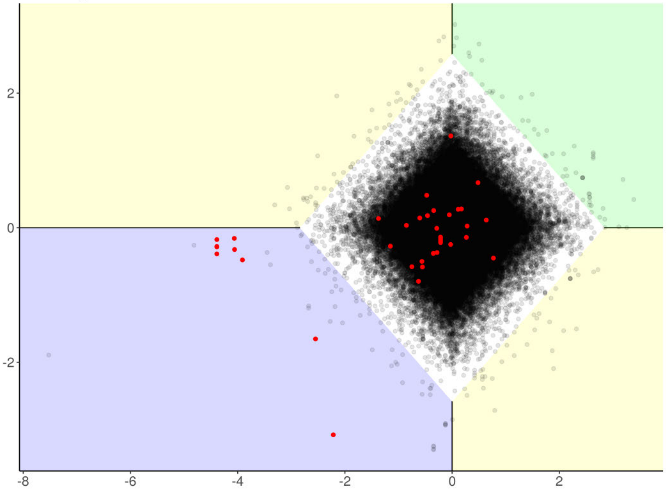

EoAB, max

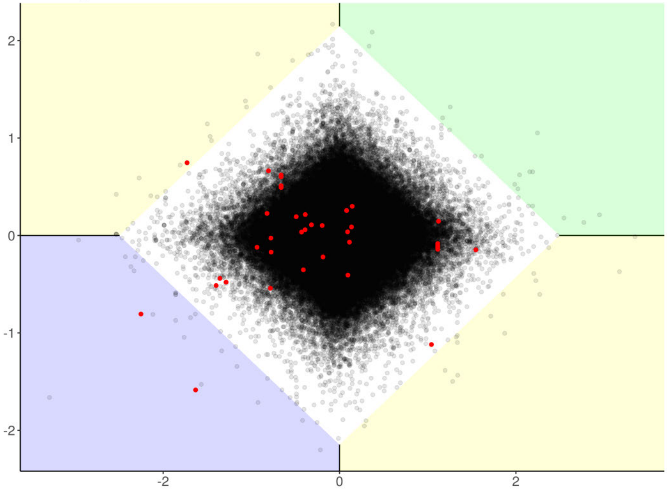

EoAB, median

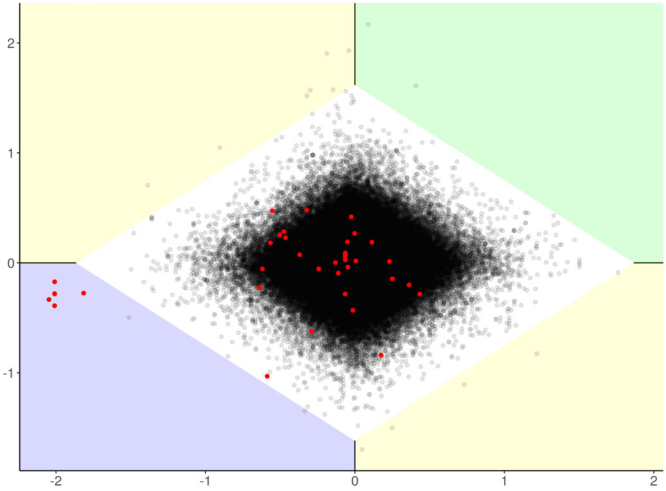

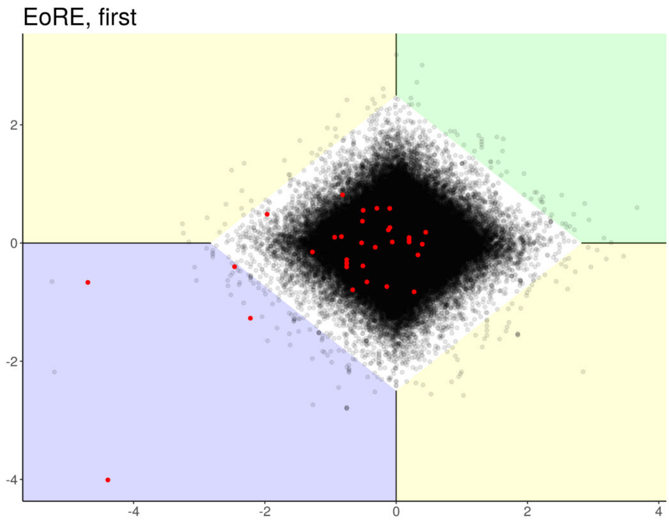
EoRE, first
2
0
-2
-4
-4
-2
0
2
4

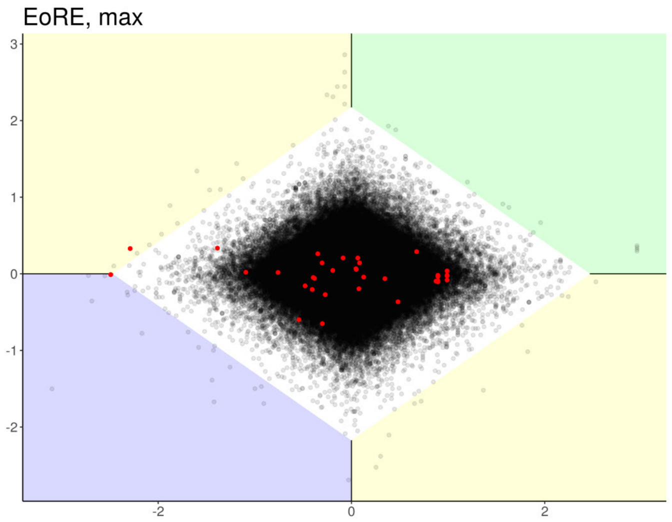
EoRE, max
3
2
1
0
-1
-2
-2
0
2

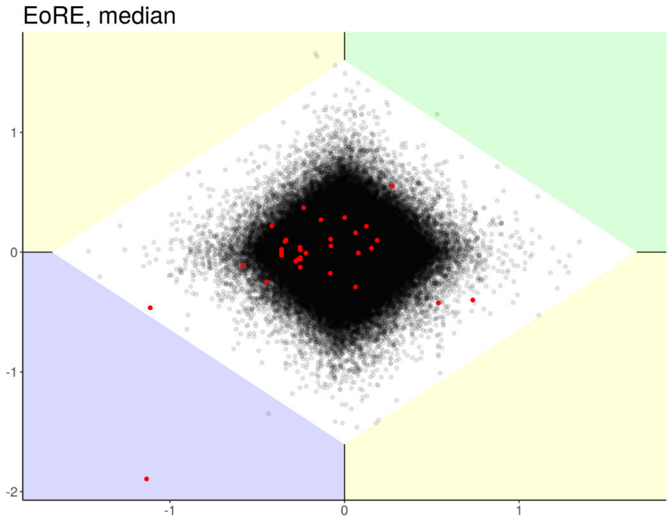
EoRE, median
1
0
-1
-2
-1
0
1

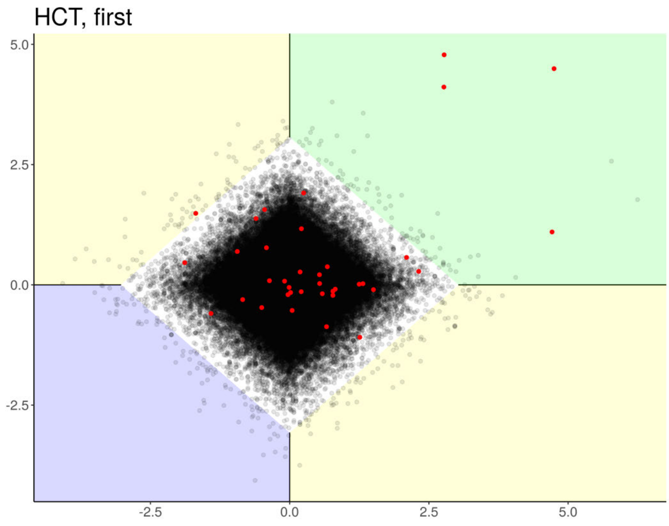
HCT, first
5.0
2.5
0.0
-2.5
-2.5
0.0
2.5
5.0

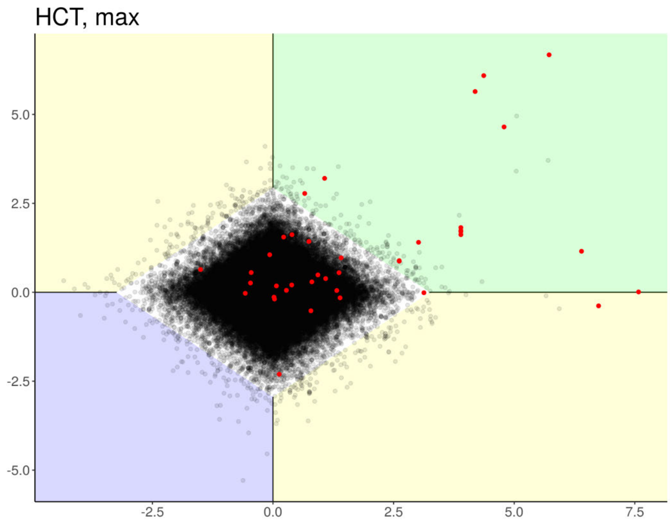
HCT, max
5.0
2.5
0.0
-2.5
-5.0
-2.5
0.0
2.5
5.0
7.5

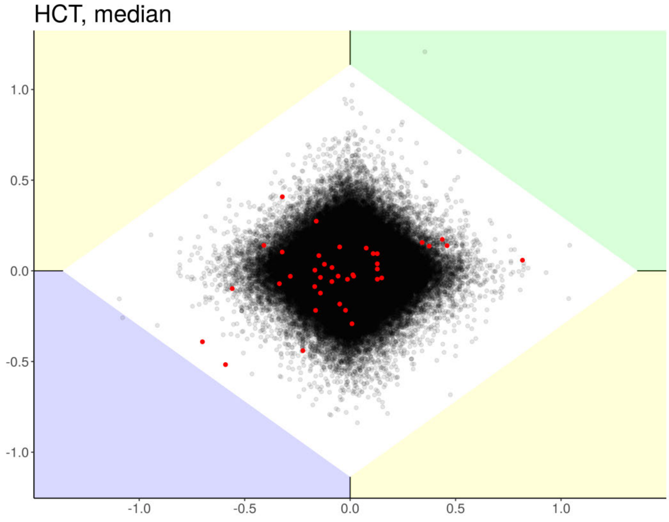
HCT, median
1.0
0.5
0.0
-0.5
-1.0
-1.0
-0.5
0.0
0.5
1.0

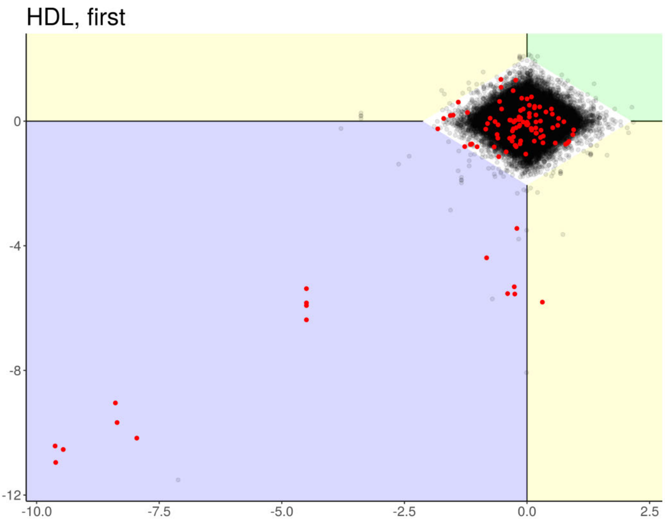
HDL, first

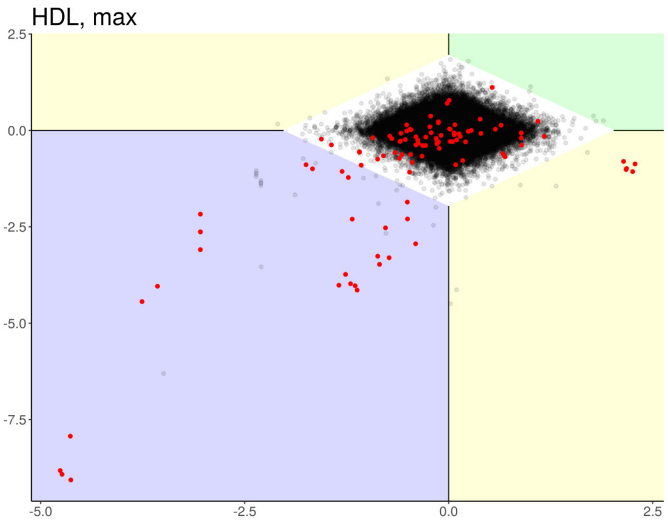
HDL, max

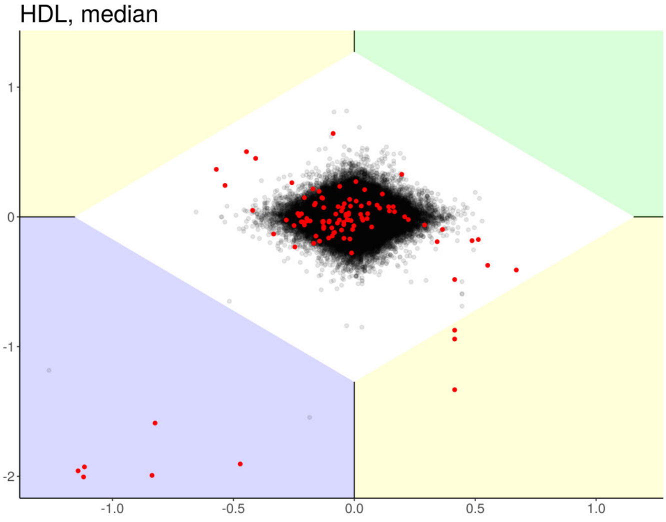
HDL, median

Hgb, first

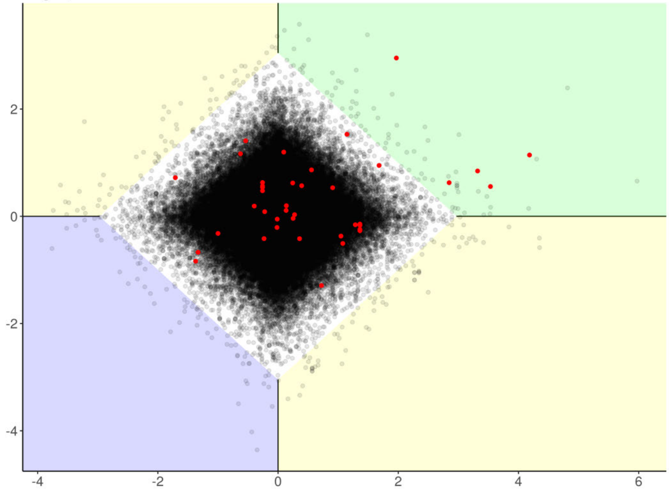

Hgb, max

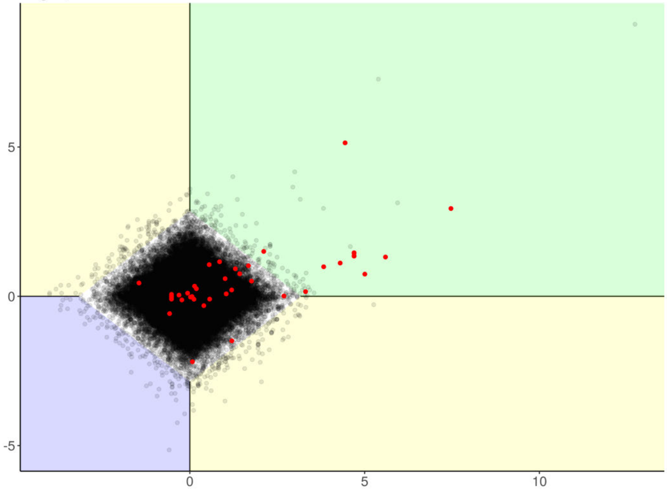

Hgb, median

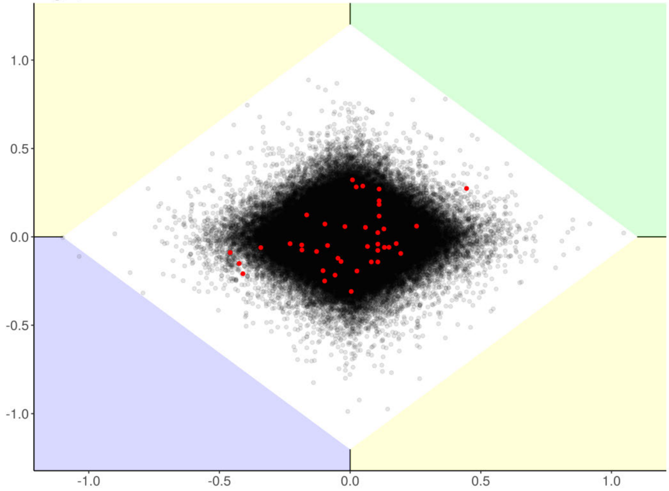

LDL, first

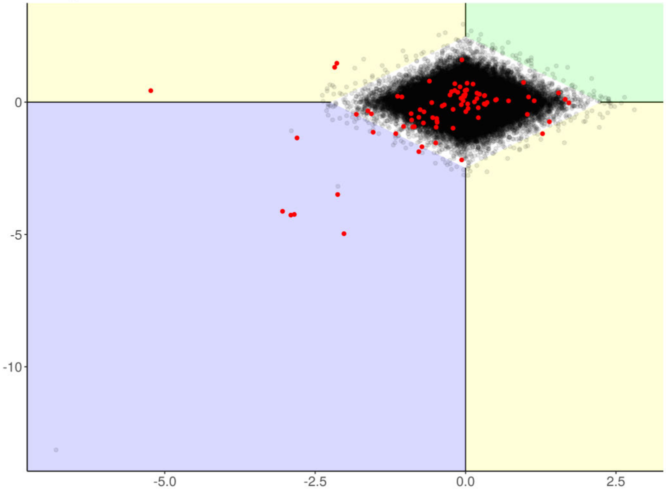

LDL, max

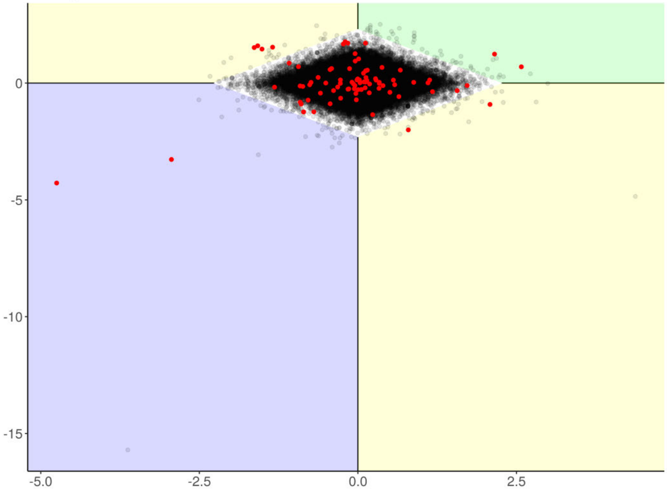

LDL, median

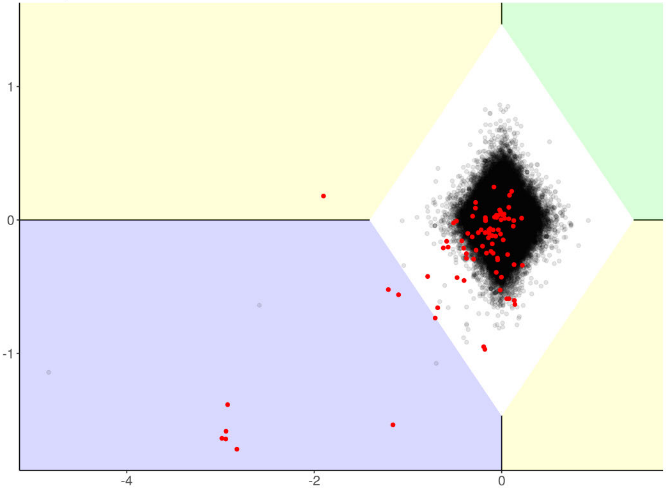

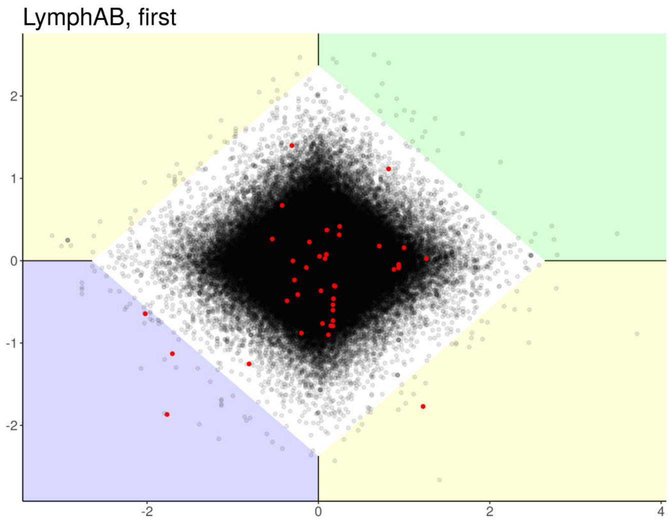
LymphAB, first
2
1
0
-1
-2
-2
0
2
4

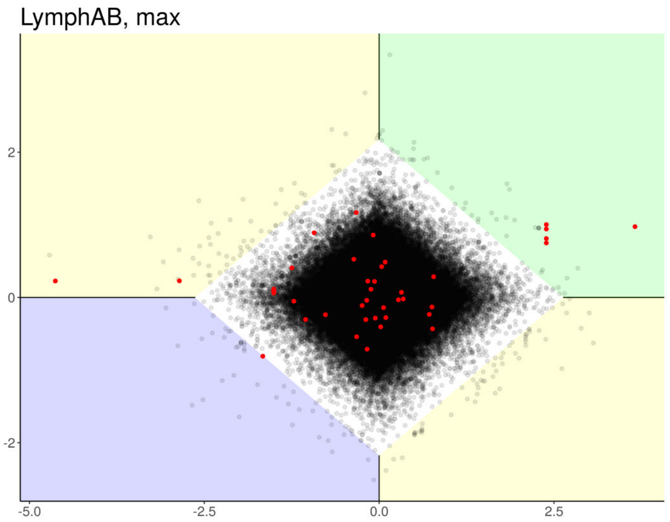
LymphAB, max
2
0
-2
-5.0
-2.5
0.0
2.5

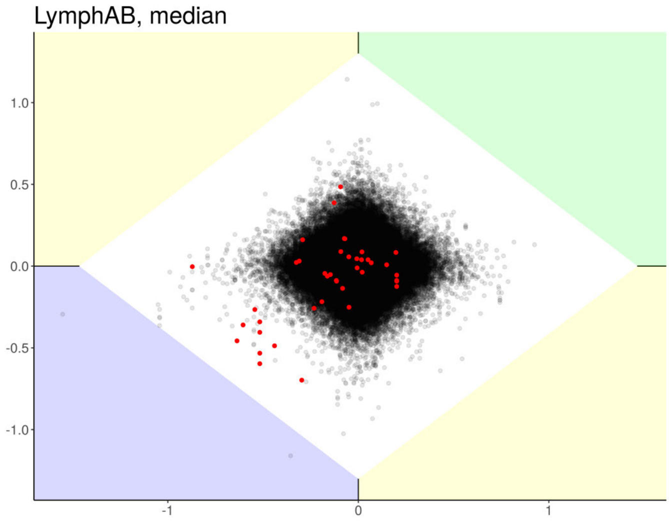
LymphAB, median
1.0
0.5
0.0
-0.5
-1.0
-1
0
1

LymphRE, first

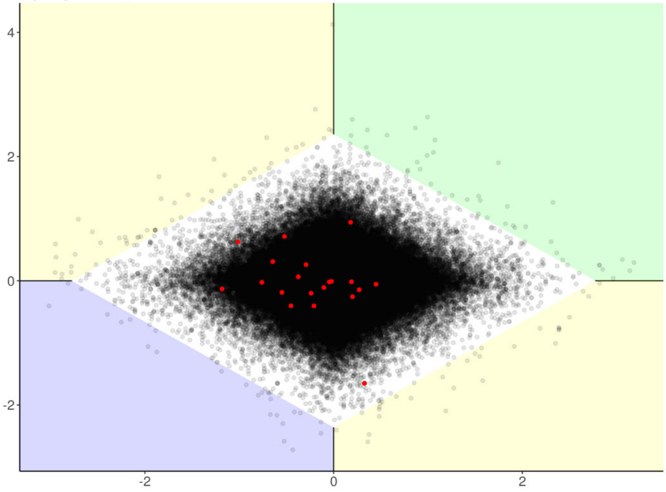

LymphRE, max

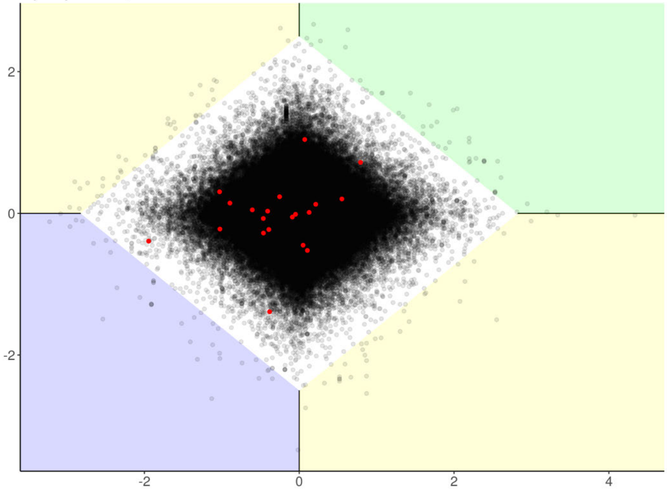

LymphRE, median

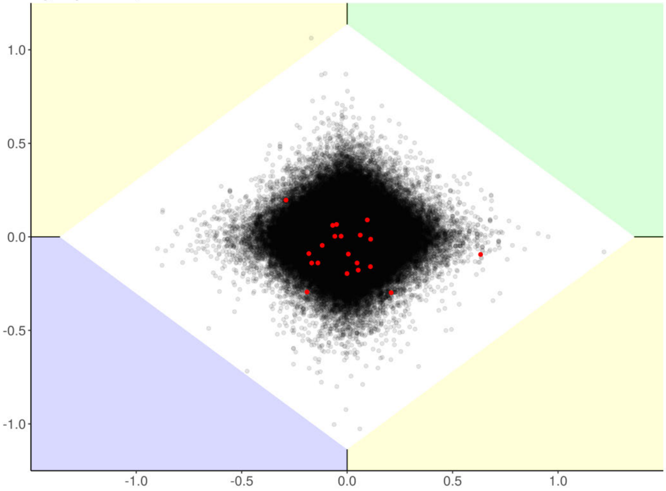

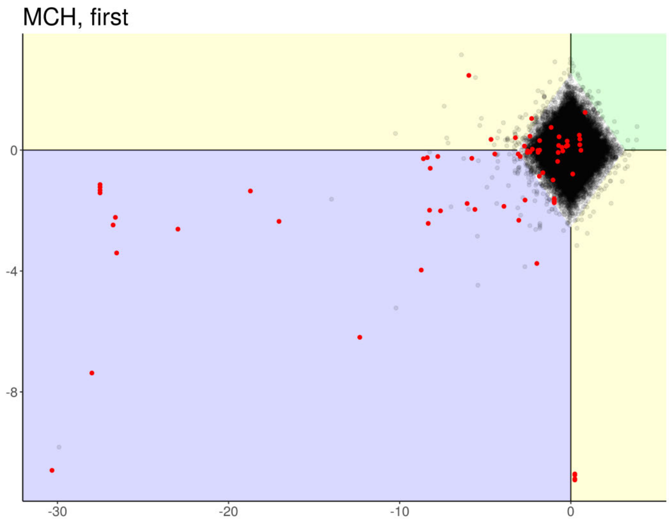
MCH, first
0
-4
-8
-30
-20
-10
0

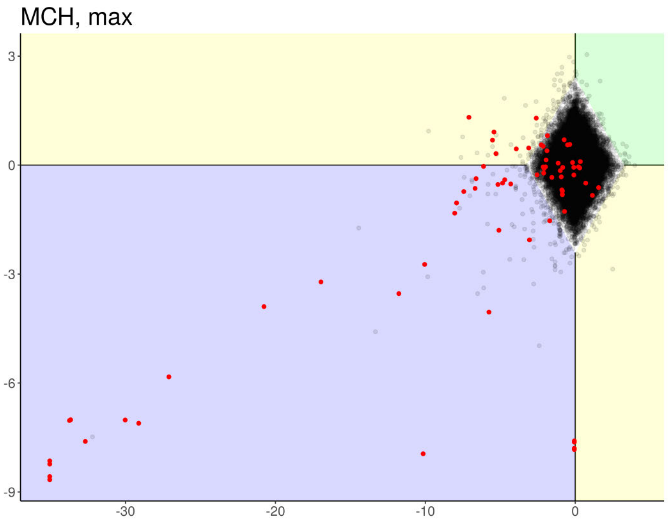
MCH, max
3
0
-3
-6
-9
-30
-20
-10
0

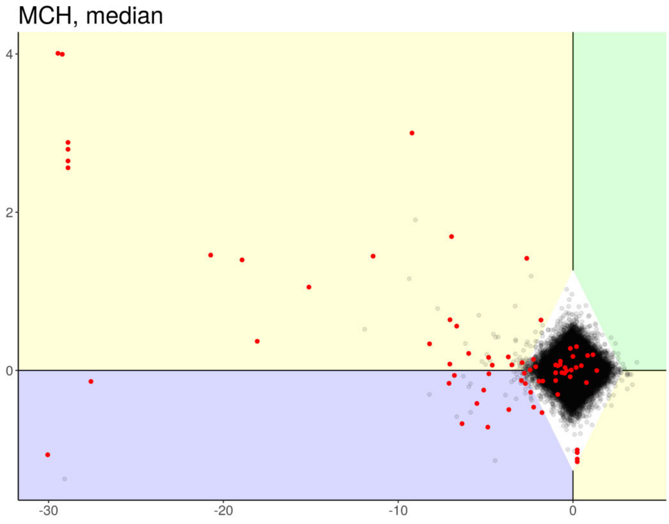
MCH, median
4
2
0
-30
-20
-10
0

MCHC, first

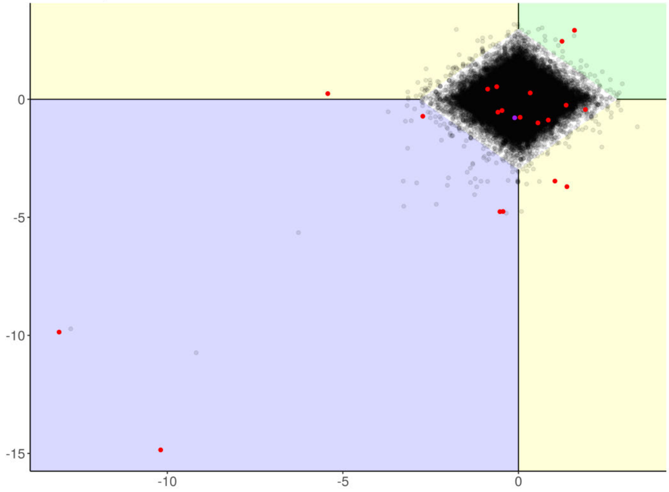

MCHC, max

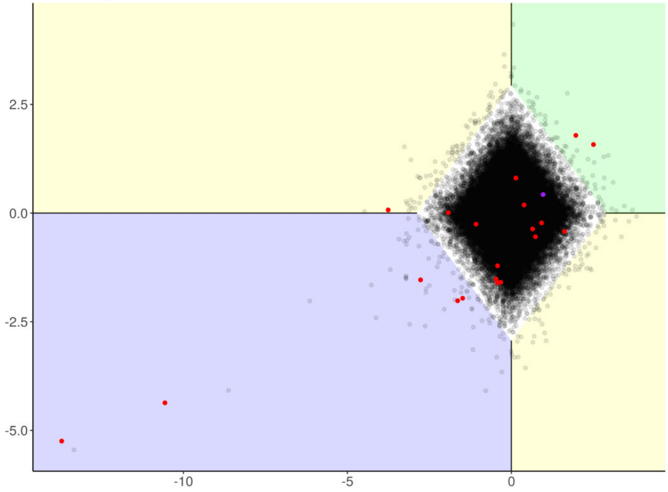

MCHC, median

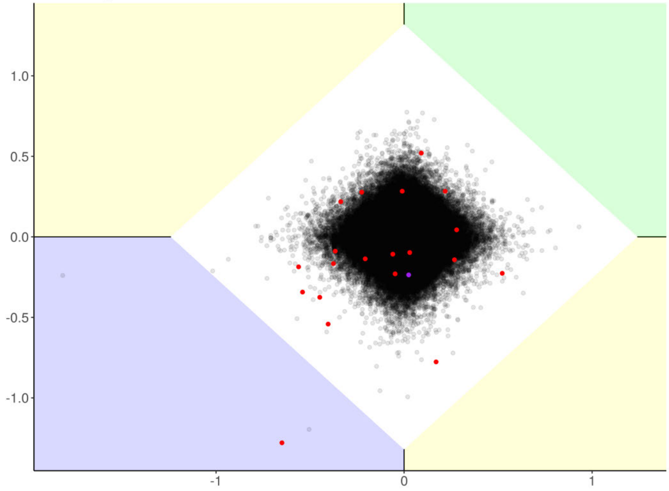

MCV, first

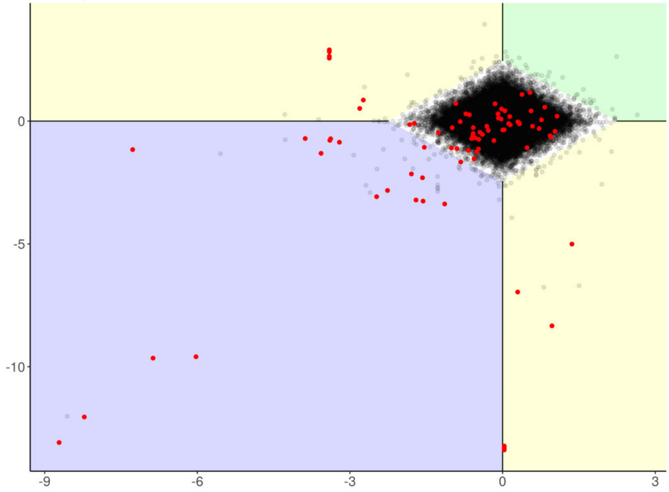

MCV, max

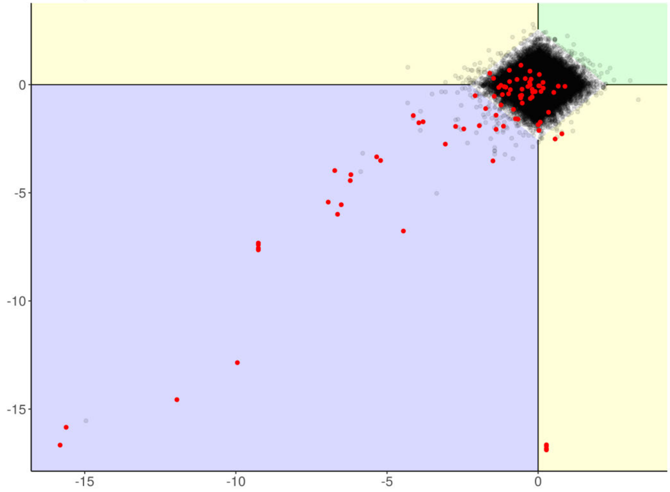

MCV, median

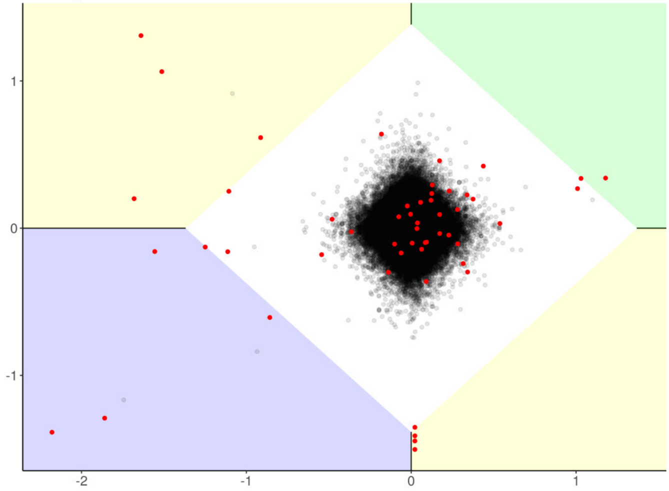

MonoAB, first

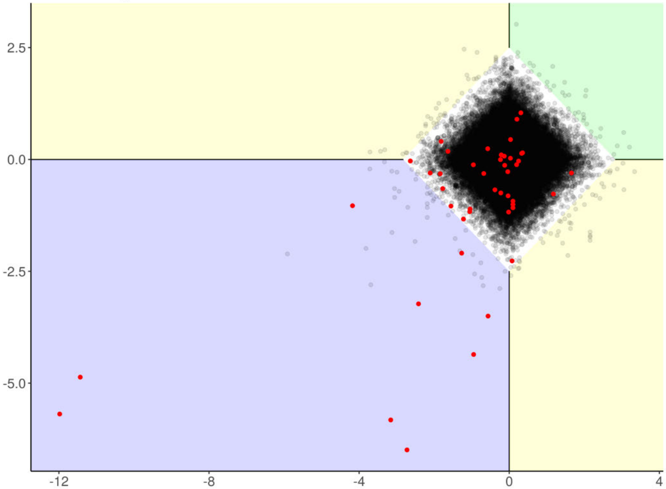

MonoAB, max

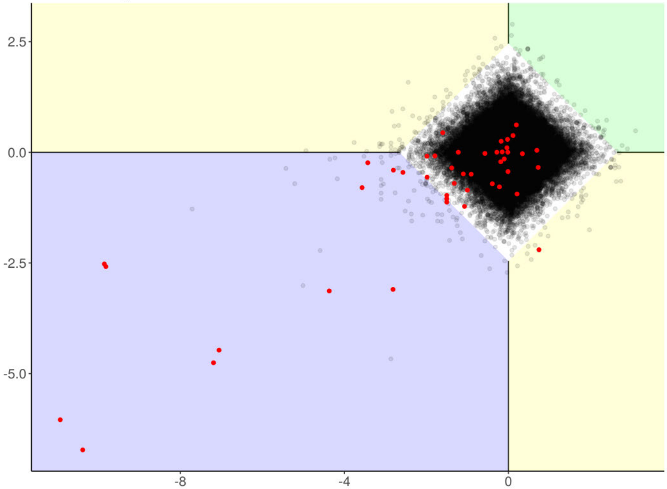

MonoAB, median

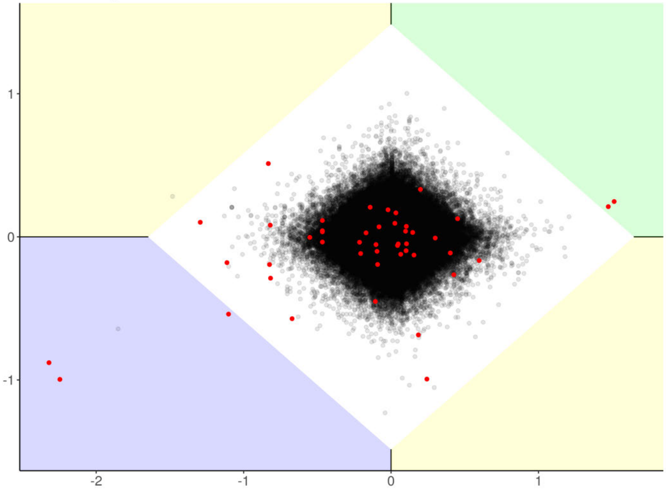

MPV, first

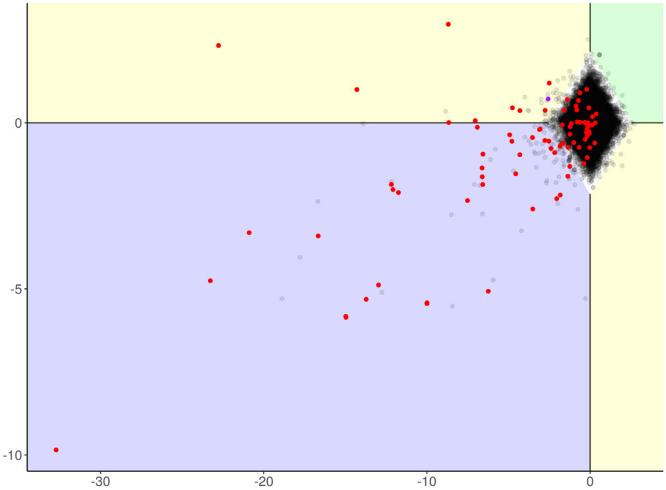

MPV, max

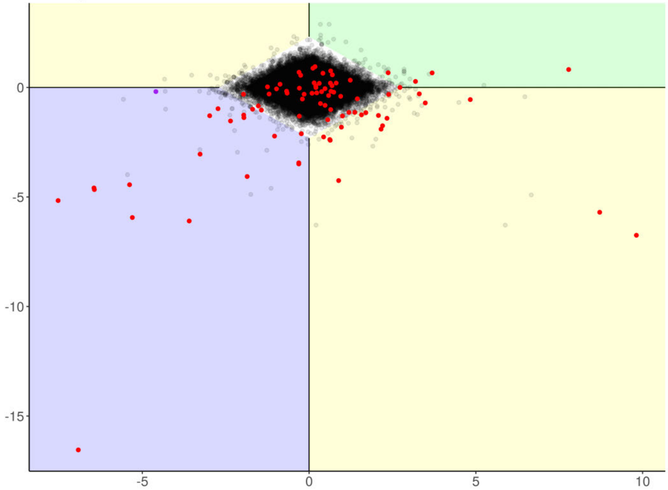

MPV, median

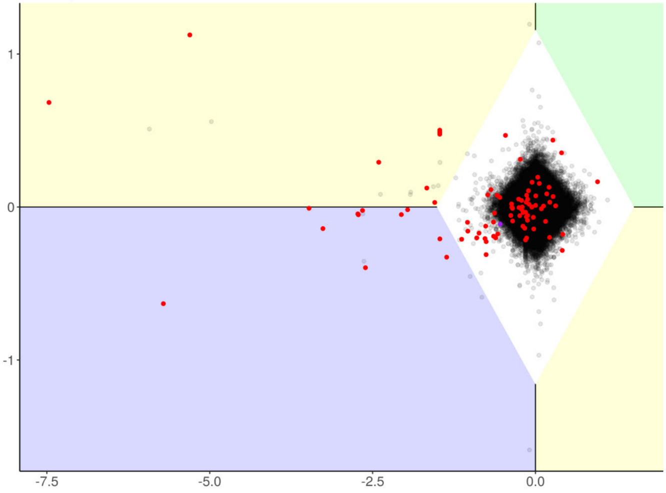

PLT, first

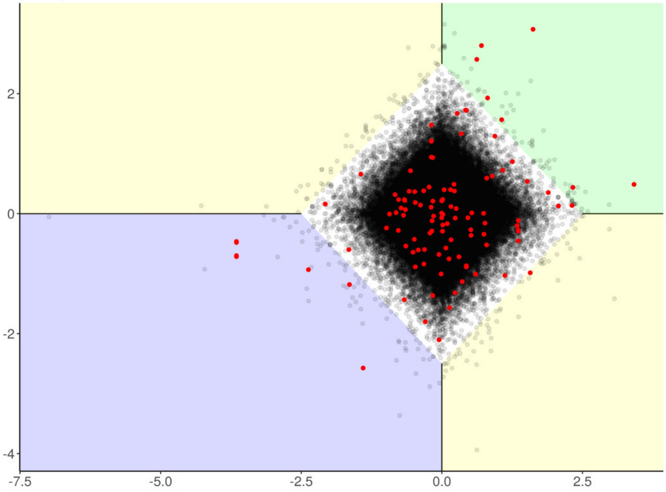

PLT, max

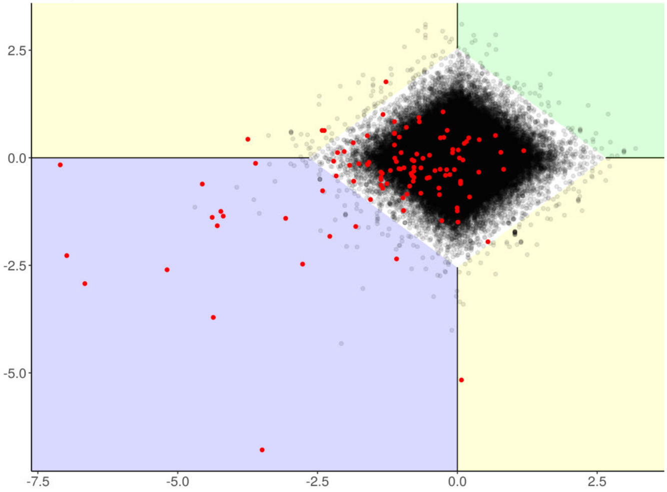

PLT, median

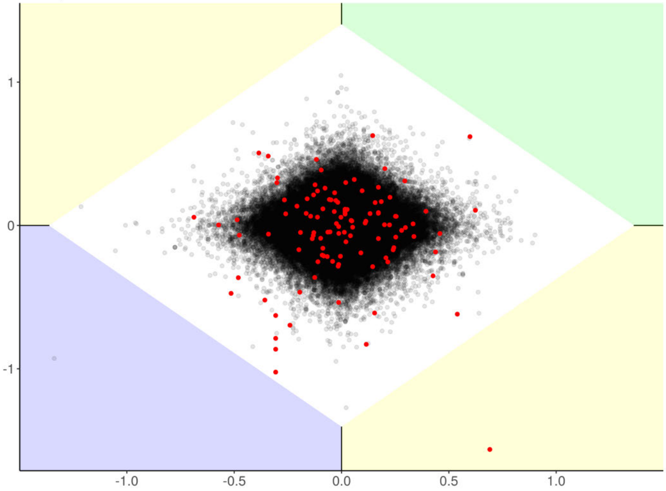

PMNAB, first

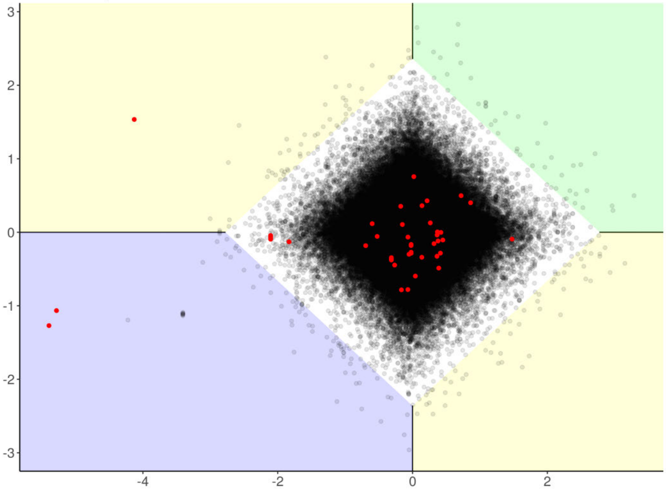

PMNAB, max

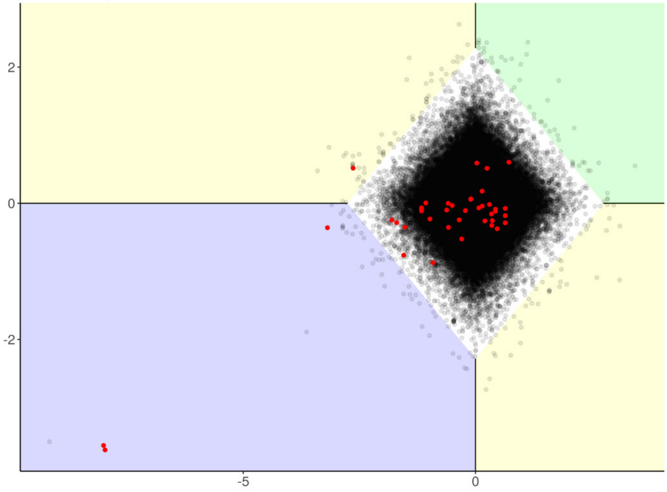

PMNAB, median

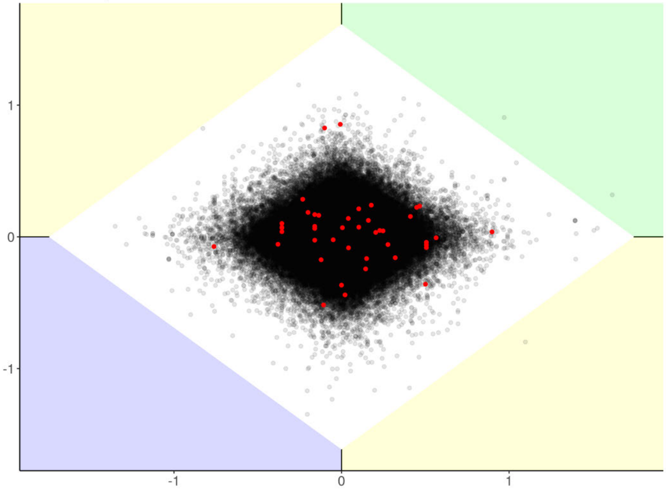

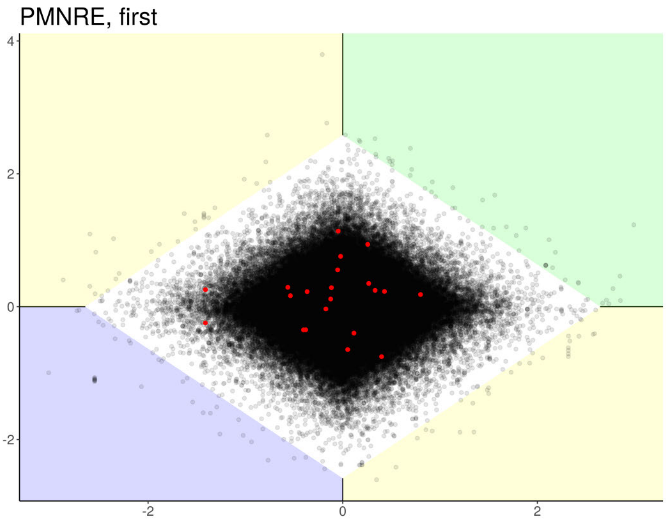
PMNRE, first
4
2
0
-2
-2
0
2

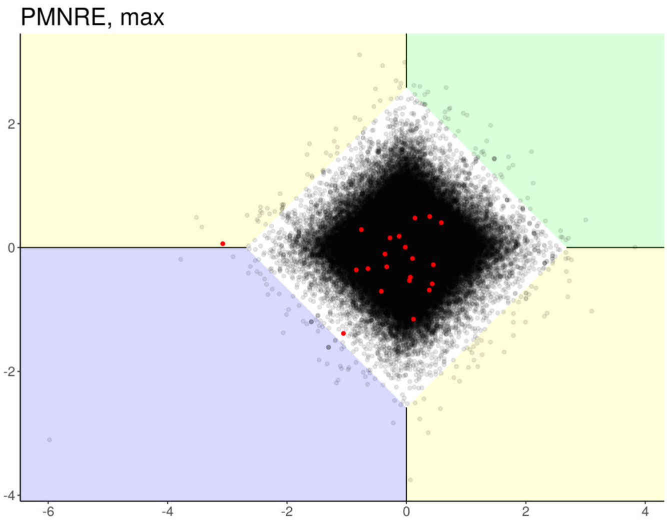
PMNRE, max
2
0
-2
-4
-6
-4
-2
0
2
4

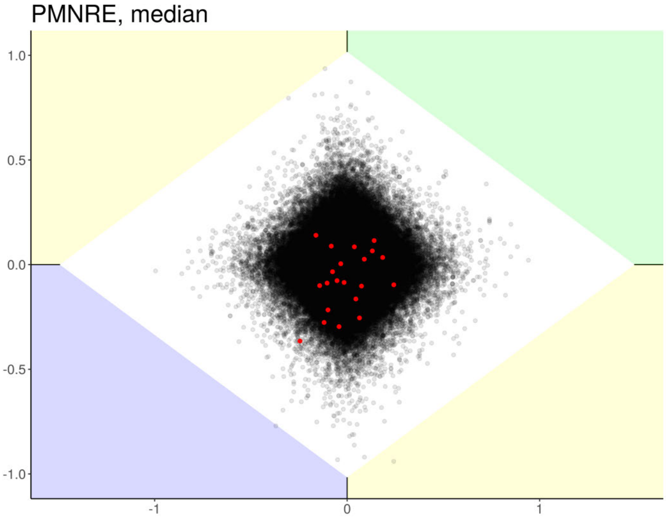
PMNRE, median
1.0
0.5
0.0
-0.5
-1.0
-1
0
1

RBC, first

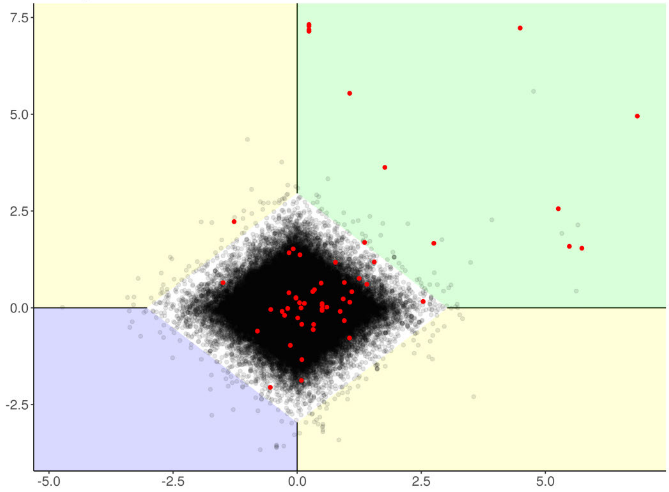

RBC, max

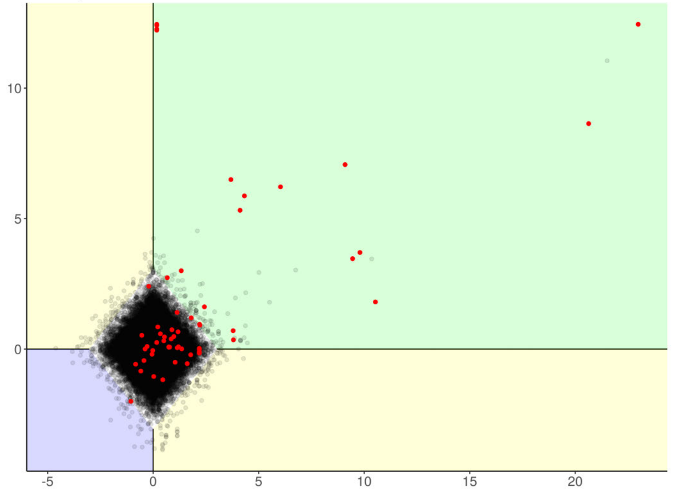

RBC, median

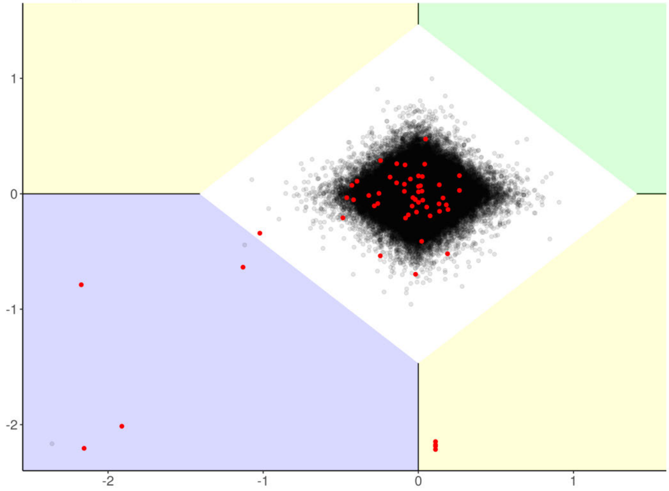

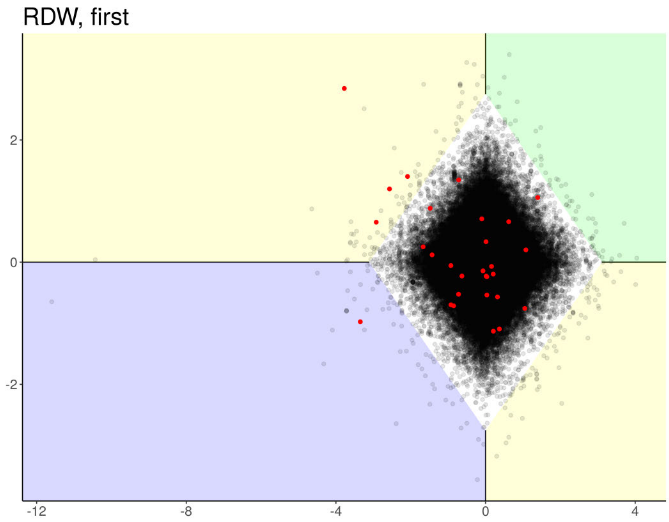
RDW, first
2
0
-2
-12
-8
-4
0
4

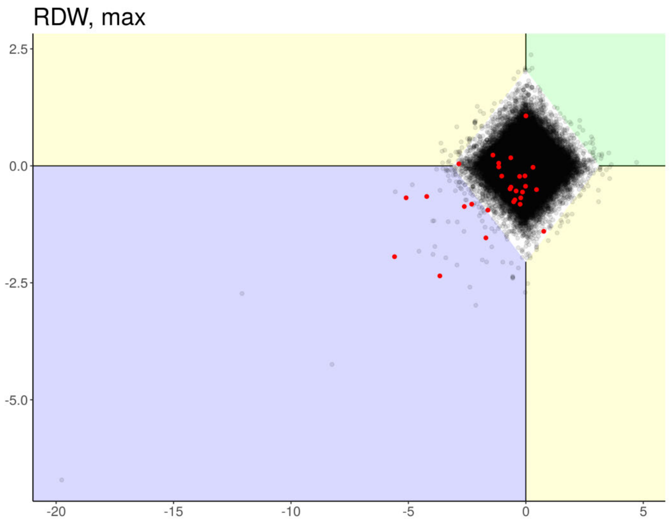
RDW, max
2.5
0.0
-2.5
-5.0
-20
-15
-10
-5
0
5

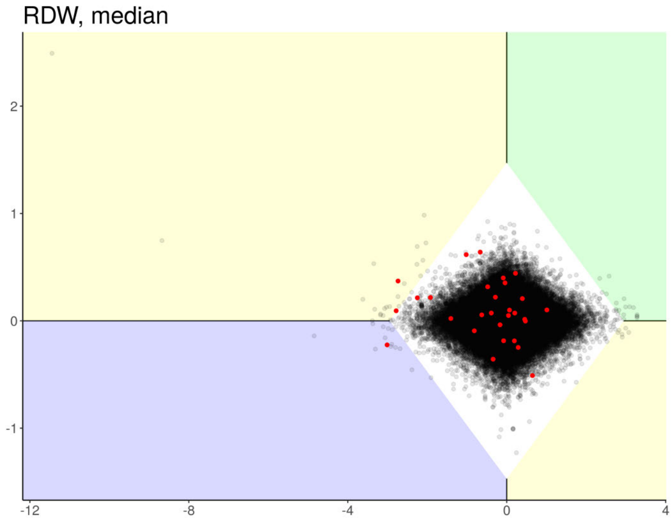
RDW, median
2
1
0
-1
-12
-8
-4
0
4

Trigs, first

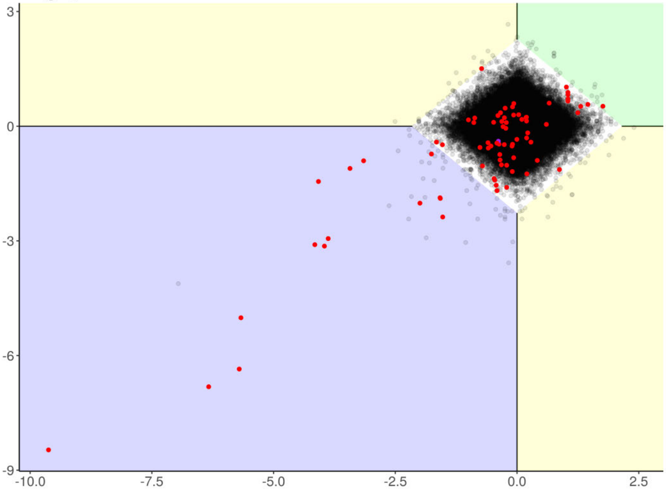

Trigs, max

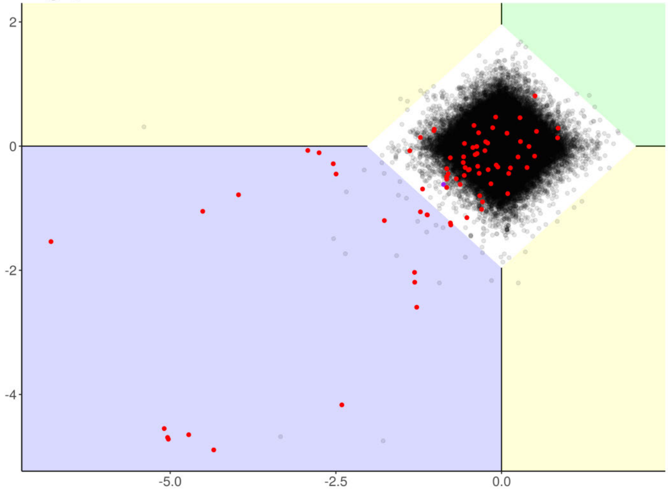

Trigs, median

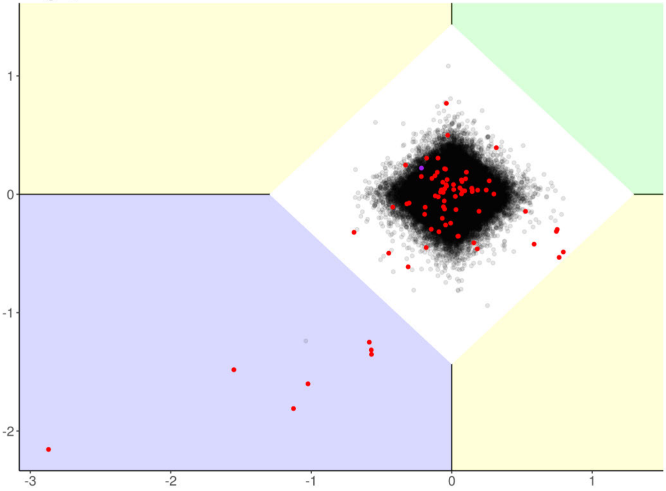

WBC, first

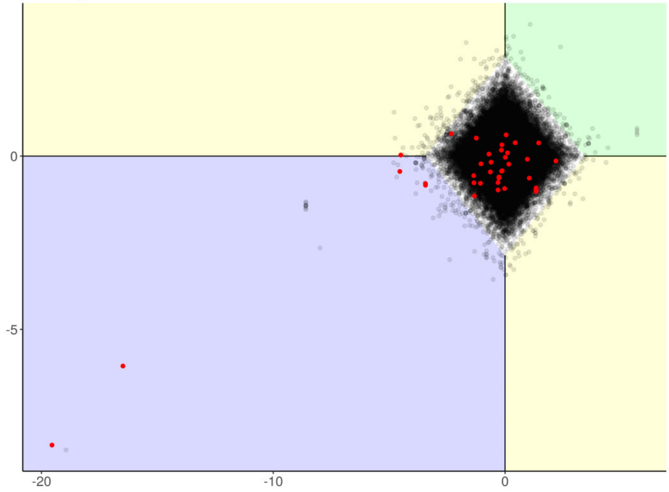

WBC, max

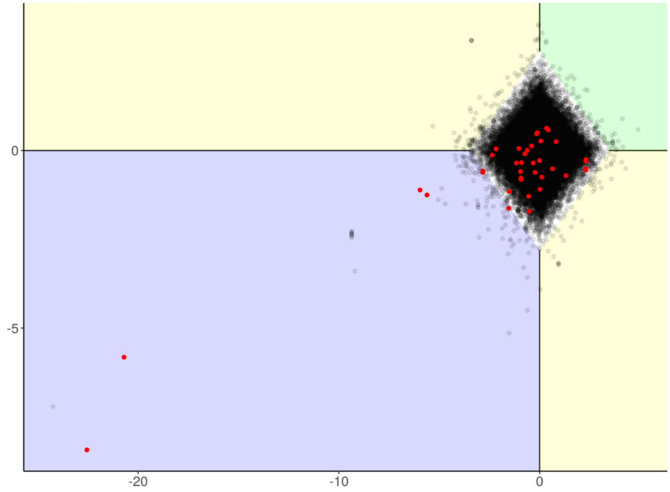

WBC, median

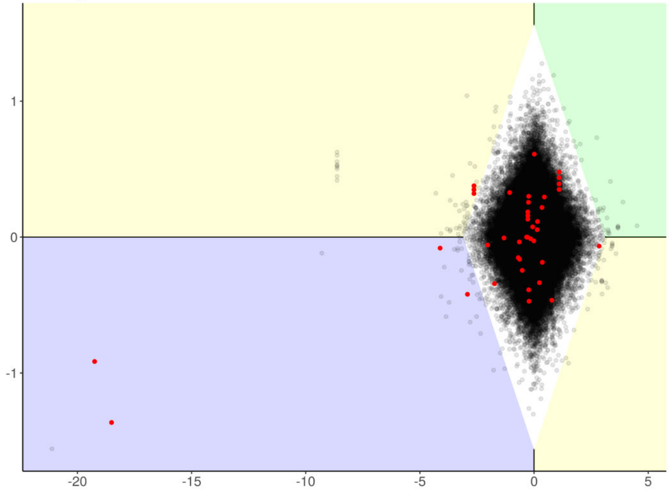

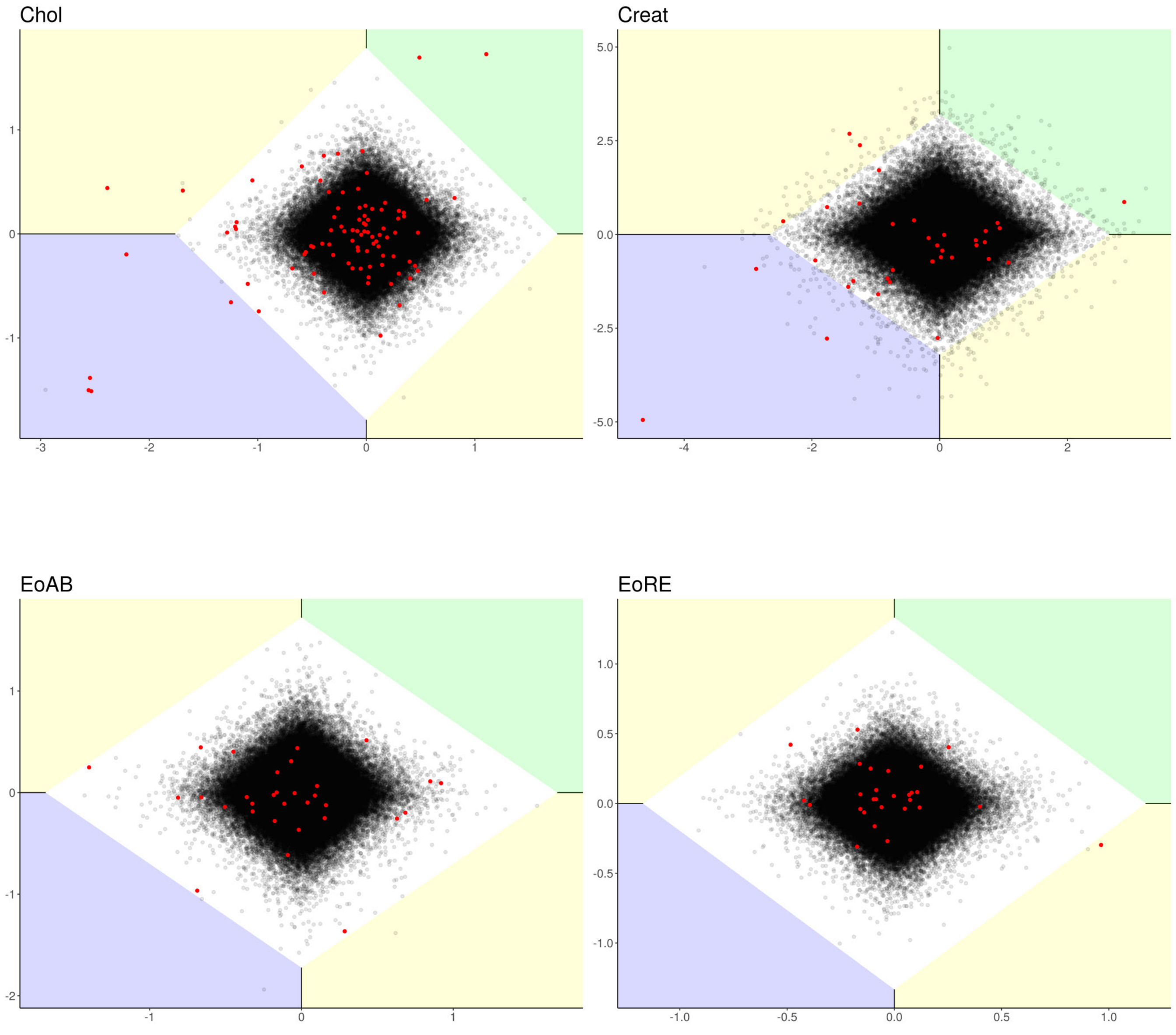
Chol
1
0
-1
-3
-2
-1
0
1
Creat
5.0
2.5
0.0
-2.5
-5.0
-4
-2
0
2
EoAB
1
0
-1
-2
-1
0
1
EoRE
1.0
0.5
0.0
-0.5
-1.0
-1.0
-0.5
0.0
0.5
1.0

HCT

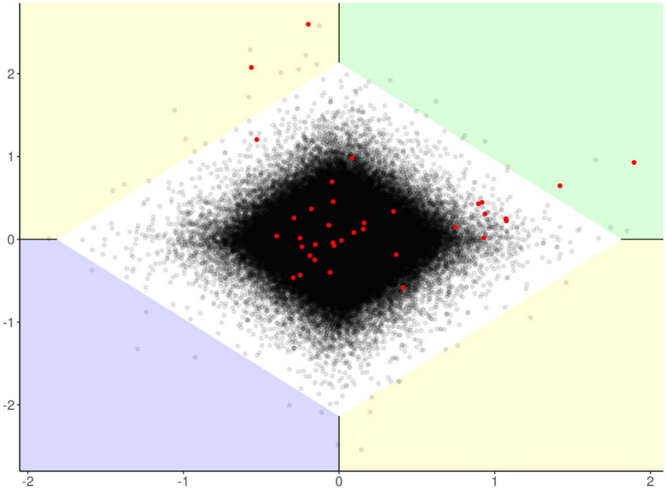

HDL

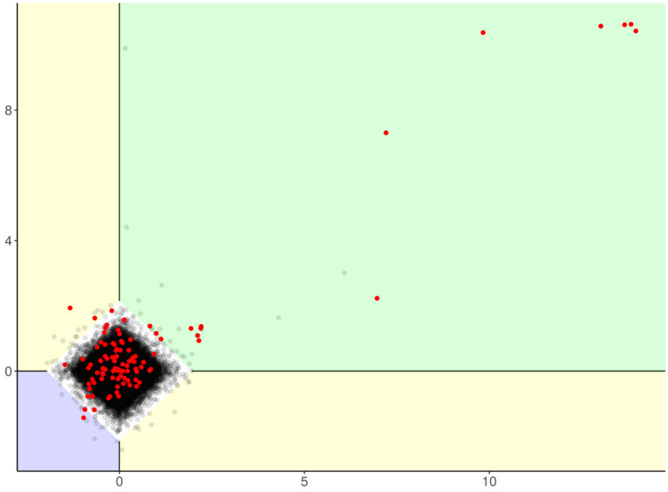

Hgb

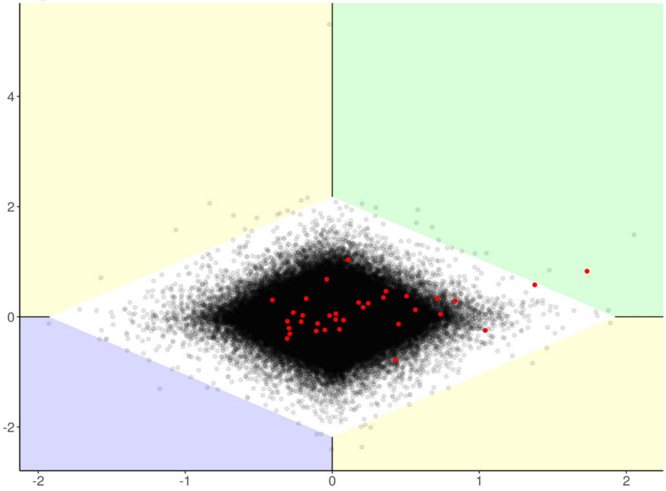

LDL

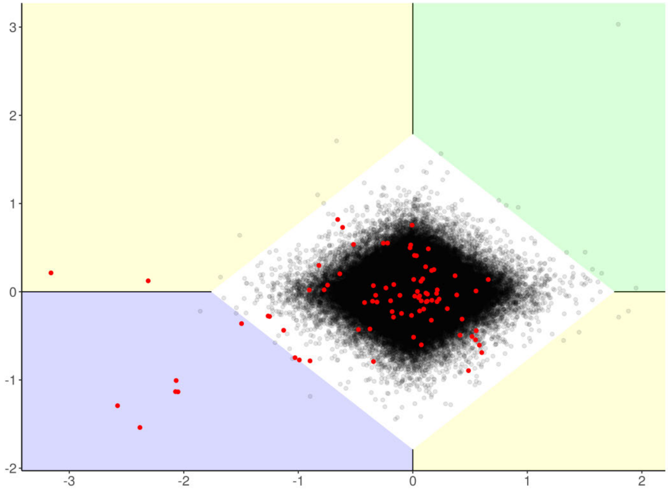

LymphAB

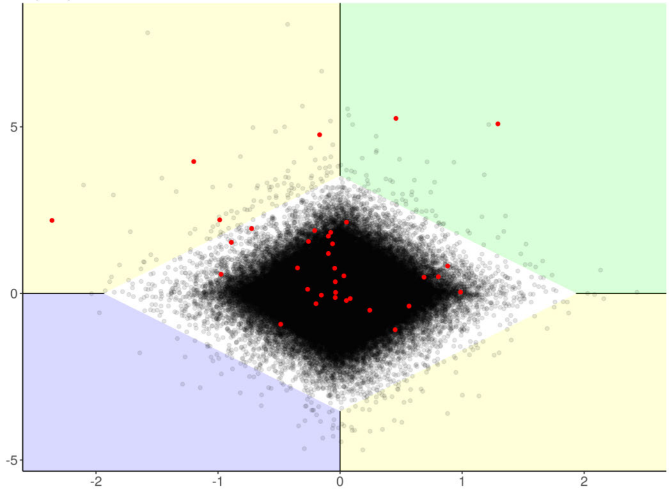

LymphRE

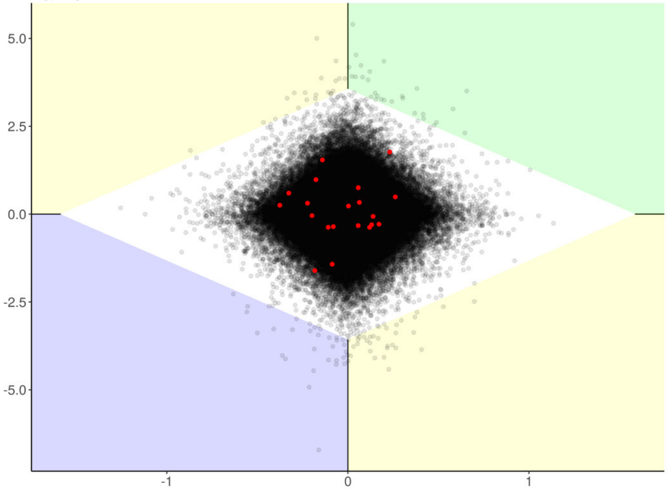

MCH

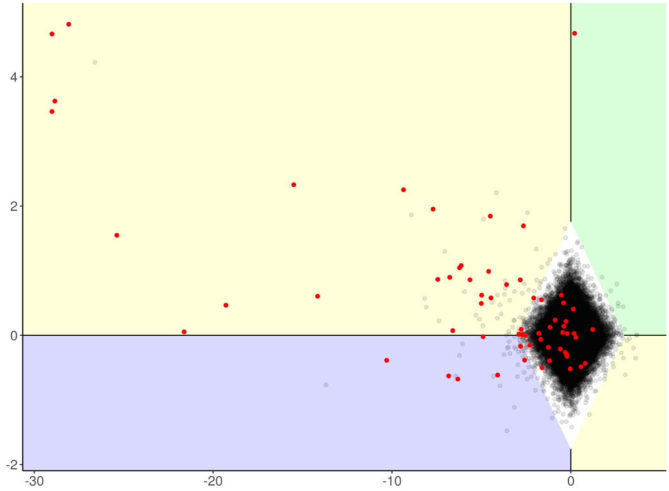

MCHC

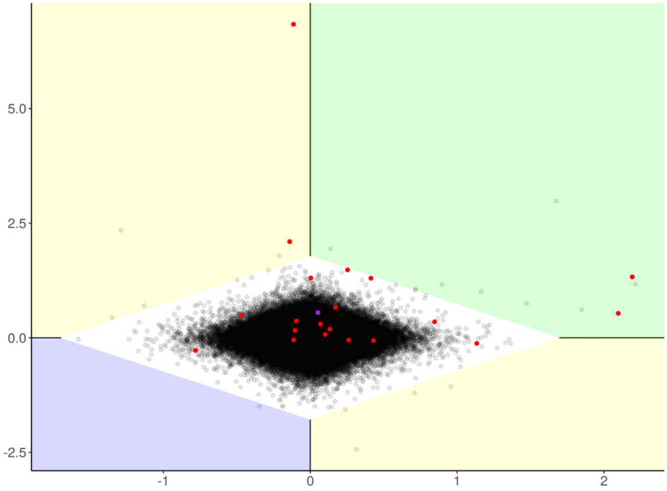

MCV

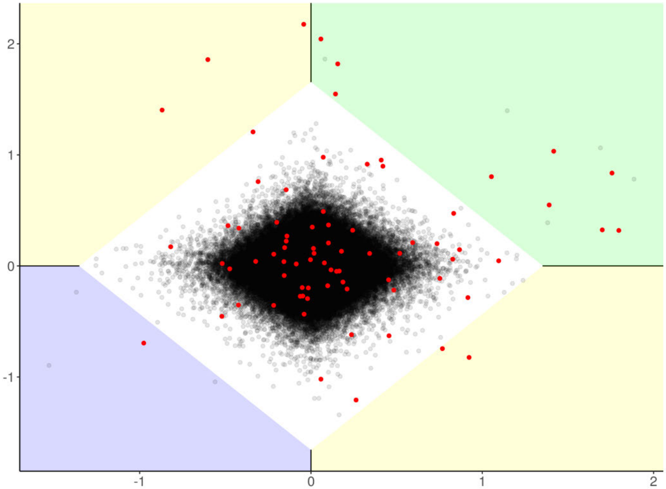

MonoAB

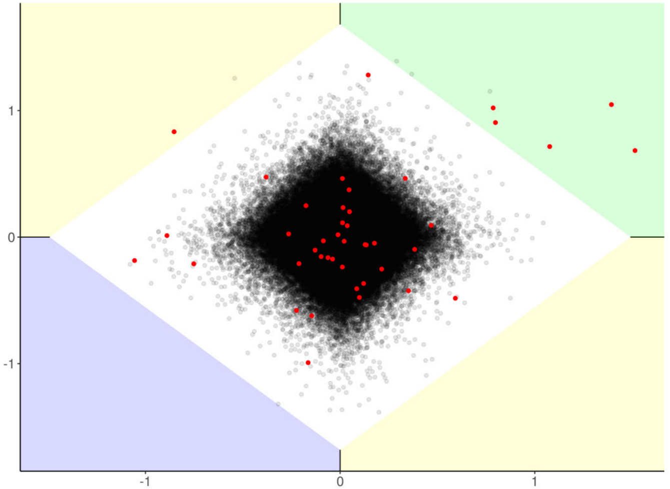

MPV

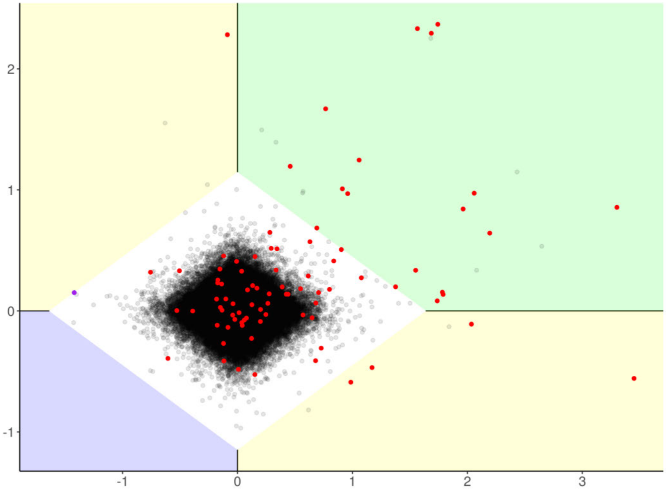

PLT

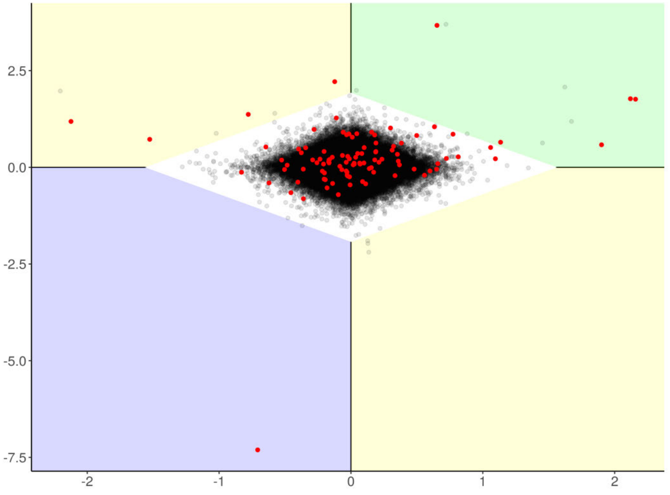

PMNAB

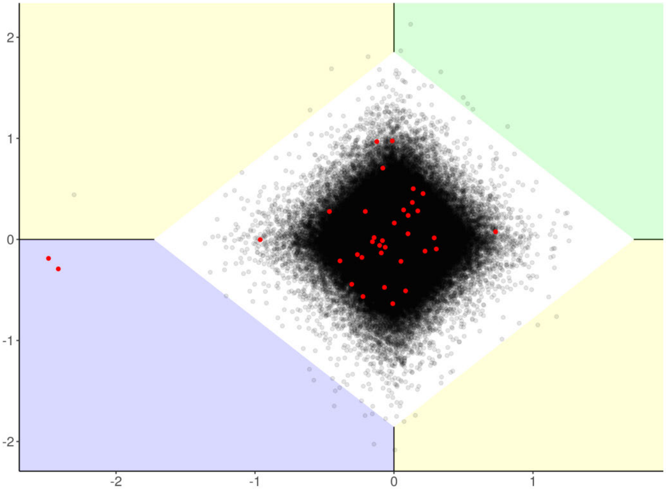

PMNRE

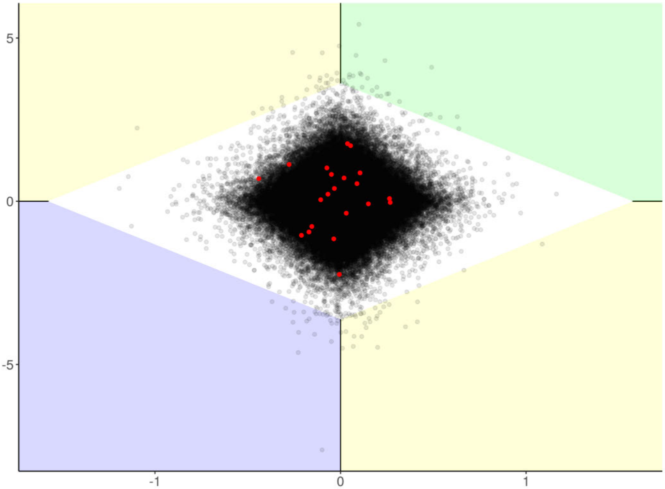

RBC

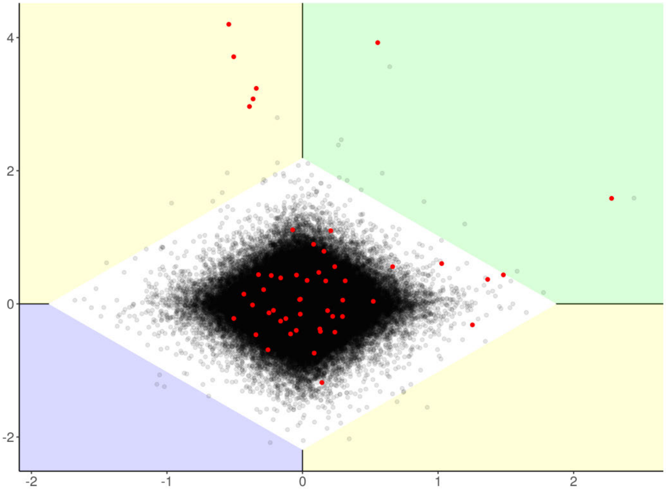

RDW

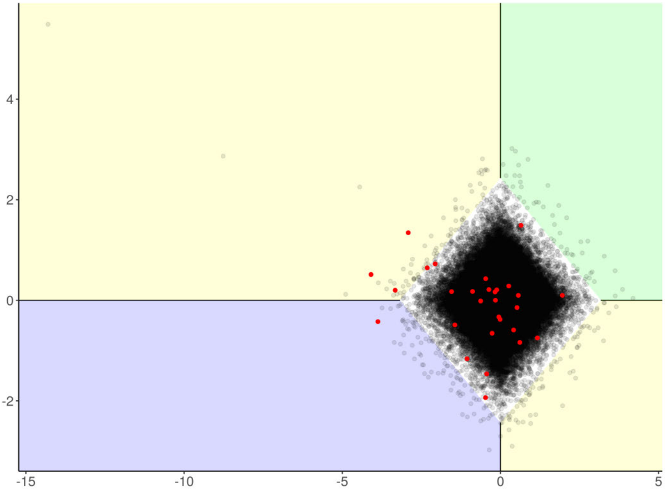

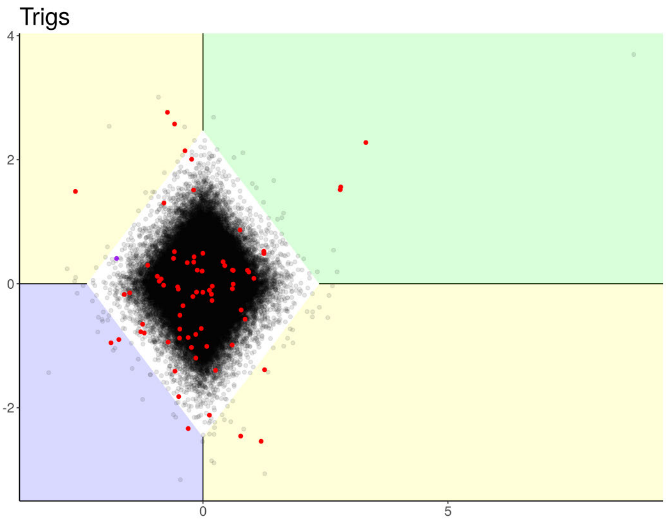
Trigs
4
2
0
-2
0
5

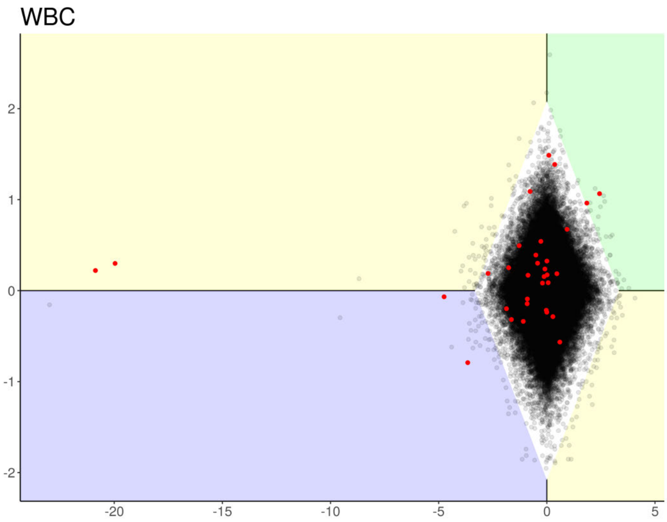
WBC
2
1
0
-1
-2
-20
-15
-10
-5
0
5
